# The Conserved SKN-1/Nrf2 Stress Response Pathway Regulates Synaptic Function in *Caenorhabditis elegans*

**DOI:** 10.1371/journal.pgen.1003354

**Published:** 2013-03-21

**Authors:** Trisha A. Staab, Trevor C. Griffen, Connor Corcoran, Oleg Evgrafov, James A. Knowles, Derek Sieburth

**Affiliations:** Zilkha Neurogenetic Institute, Keck School of Medicine, University of Southern California, Los Angeles, California, United States of America; University of California San Francisco, United States of America

## Abstract

The Nrf family of transcription factors plays a critical role in mediating adaptive responses to cellular stress and defends against neurodegeneration, aging, and cancer. Here, we report a novel role for the *Caenorhabditis elegans* Nrf homolog SKN-1 in regulating synaptic transmission at neuromuscular junctions (NMJs). Activation of SKN-1, either by acute pharmacological treatment with the mitochondrial toxin sodium arsenite or by mutations that cause constitutive SKN-1 activation, results in defects in neuromuscular function. Additionally, elimination of the conserved WD40 repeat protein WDR-23, a principal negative regulator of SKN-1, results in impaired locomotion and synaptic vesicle and neuropeptide release from cholinergic motor axons. Mutations that abolish *skn-1* activity restore normal neuromuscular function to *wdr-23* mutants and animals treated with toxin. We show that negative regulation of SKN-1 by WDR-23 in the intestine, but not at neuromuscular junctions, is necessary and sufficient for proper neuromuscular function. WDR-23 isoforms differentially localize to the outer membranes of mitochondria and to nuclei, and the effects of WDR-23 on neuromuscular function are dependent on its interaction with cullin E3 ubiquitin ligase. Finally, whole-transcriptome RNA sequencing of *wdr-23* mutants reveals an increase in the expression of known SKN-1/Nrf2-regulated stress-response genes, as well as neurotransmission genes not previously implicated in SKN-1/Nrf2 responses. Together, our results indicate that SKN-1/Nrf2 activation may be a mechanism through which cellular stress, detected in one tissue, affects cellular function of a distal tissue through endocrine signaling. These results provide insight into how SKN-1/Nrf2 might protect the nervous system from damage in response to oxidative stress.

## Author Summary

Transcriptional programs control cellular responses in the face of environmental stress, such as dietary restriction, hypoxia, or oxidative stress. Furthermore, in order to promote survival of the organism in response to insult, communication between tissues must be established. Using the model system *C. elegans*, we investigate functional changes in the nervous system mediated by the transcription factor SKN-1. We establish that activation of SKN-1, either genetically or through exposure to the mitochondrial toxin arsenite, results in locomotion changes that take place at the neuromuscular junction. Furthermore, these changes in the nervous system are brought about through signaling from the intestine. Lastly, we use whole-transcriptome RNA sequencing to identify new transcriptional targets of SKN-1 that might be affecting locomotory behavior. Our results indicate that neuronal function can be regulated at the level of the synapse in response to environmental stress.

## Introduction

To prevent cellular damage caused by environmental stresses, organisms have developed defense mechanisms at the cellular level which ultimately result in system-wide protective changes. Oxidative stress is generated in part by an increase in reactive oxygen species and has been associated with increased protein and lipid damage and reduced mitochondrial function. Elevated levels of reactive oxygen species and a concomitant decrease in antioxidants have been linked to aging, neurodegeneration and diabetes [1], [2]. How organisms are able to coordinate an appropriate, systemic response to prevent damage caused by oxidative stress is not well understood.

The Nrf (nuclear factor E2-related factor) family of transcription factors, homologous to CNC [3] and SKN-1 [4] in *Drosophila melanogaster* and *Caenorhabditis elegans*, respectively, is activated in response to heightened oxidative stress or drugs (xenobiotics) [1], [5]. When activated, Nrf proteins move into the nucleus where they bind the antioxidant response element, or ARE, in enhancer regions of target genes [1], [5]. Nrf nuclear translocation results in the expression of a host of phase II detoxificants and antioxidants, including glutathione-synthesizing enzymes, thioredoxins, and proteasomal subunits; together, these enzymes are proposed to collectively mitigate the harmful effects of reactive oxygen species [6]–[9].

Nrf family members promote systemic changes in many tissues which together enhance organismal survival and longevity [1], [5]. For example, in mice cortical cultures, activation of neuronal Nrf2 results in ARE-mediated transcription of many genes, including the excitatory amino acid transporter 3 (EAAT3), which allows neurons to import the antioxidant glutathione [10]; at the same time, Nrf2 activation in astrocytes is proposed to confer protection to neurons by increasing glutathione release [6], [8], [11]. In another example, disruption of Nrf-1 in mice results in embryonic lethality due to a cell non-autonomous defect in erythropoiesis [12]. Similarly, in *C. elegans*, SKN-1 functions in a pair of neurons to promote longevity through endocrine signaling that affects distal tissues [13], [14]. Thus, SKN-1/Nrf signaling acts cell-autonomously, as well as non-autonomously, to coordinate adaptive responses in response to stress.

Due in part to the high metabolic demands required for neuronal function, the nervous system is particularly susceptible to oxidative damage [1], [2]. Reactive oxygen species cause protein aggregation, mitochondrial dysfunction, altered synaptic transmission, and are associated with neurodegeneration [2], [15]. In addition to promoting neuron survival, Nrf2 activation improves cognitive performance following traumatic brain injury [16], raising the possibility that Nrf2 might regulate aspects of neuronal function. Gene expression analyses across multiple species indicate that, in addition to antioxidants, activation of SKN-1/Nrf2 also increases the expression of genes involved in calcium homeostasis, synaptic function, and signaling [8], [17], [18]; however, whether SKN-1/Nrf2 transcriptional programs directly regulate neuronal function is not known.

In *C. elegans*, SKN-1 activity is negatively regulated by the WD40 repeat protein WDR-23. WDR-23 contains seven WD repeats and is proposed to negatively regulate SKN-1 activity by targeting SKN-1 for degradation [19], [20]. *wdr-23* mutants are resistant to oxidative stress and display increased expression of SKN-1 transcriptional targets [19], [20]. This has led to the model that oxidative stress stabilizes nuclear SKN-1 by disrupting WDR-23-mediated SKN-1 degradation. WDR-23 is highly conserved, sharing 41% amino acid similarity with its human ortholog, WDR23. WDR23 is a component of the DDB1-CUL4 ubiquitin ligase complex and is proposed to act as a substrate specificity subunit that targets proteins for degradation [21]–[23].

Here, we demonstrate that WDR-23 promotes synaptic transmission at neuromuscular junctions in *C. elegans* by negatively regulating *skn-1*. We show that mutants lacking *wdr-23* have defects in locomotion and neurotransmitter secretion. We provide functional evidence that the effects of *wdr-23* on synaptic function are entirely mediated by *skn-1*, and activation of SKN-1 by mitochondrial stress can mimic the effects of *wdr-23* on synaptic function. WDR-23 acts cell non-autonomously in the intestine, localizes to the nucleus as well as to mitochondria, and loss of *wdr-23* results in altered expression of over 2000 genes. Our results support a model whereby presynaptic function is negatively regulated by a SKN-1 dependent transcriptional pathway in the intestine in response to stress.

## Results

### *wdr-23* Mutants Have Altered Locomotion Rates

**Figure 1:**
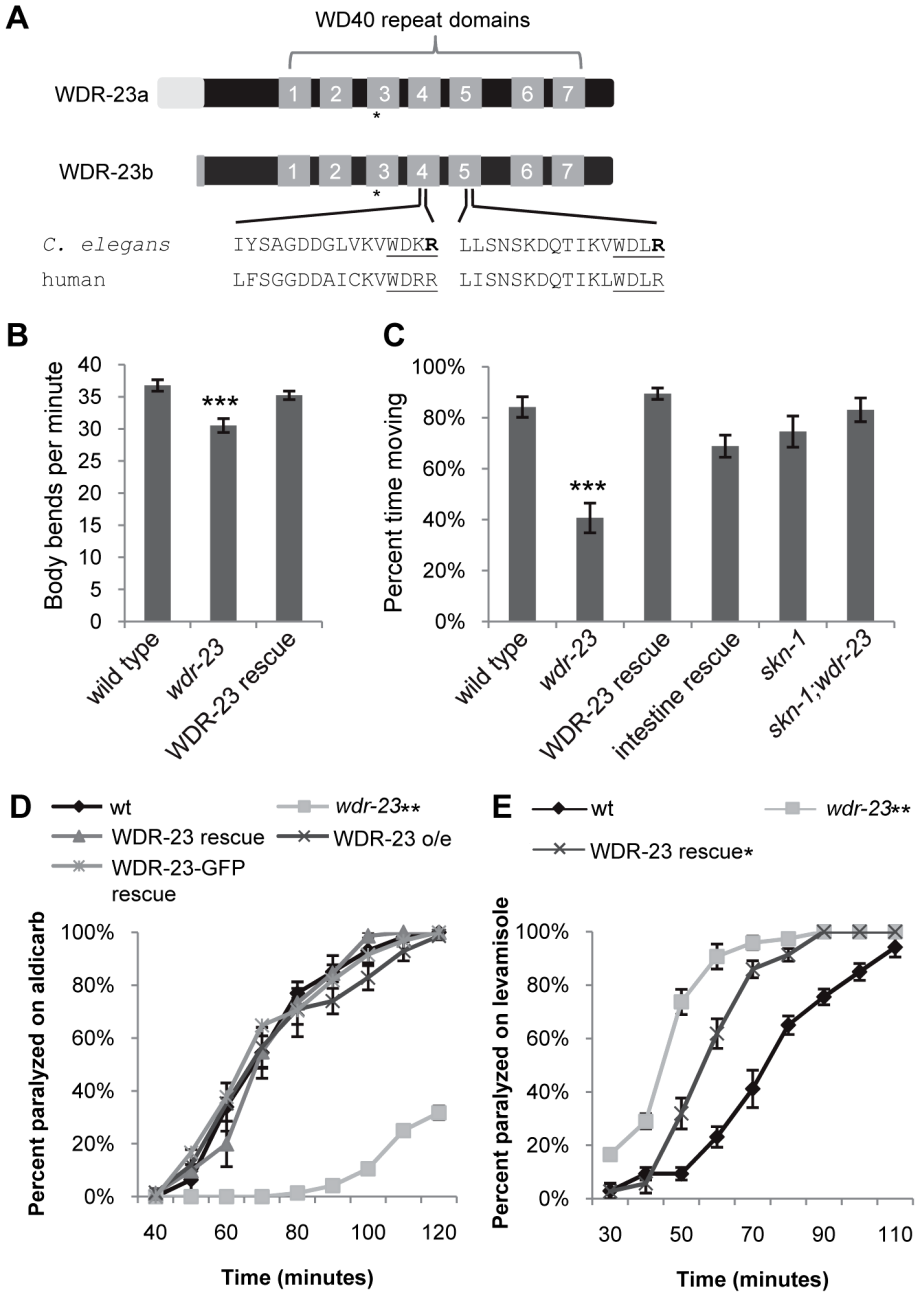
*wdr-23* mutants display locomotion defects. (A) Schematic of WDR-23 isoforms used in this study. Asterisk denotes location of premature stop in the *wdr-23(tm1817)* allele. The seven WD40 repeat domains and the conservation of the DDB1 binding sequences are identified. The Arg (R) residues which were mutated to His (H) to create WDR-23(DxR) are indicated in bold font. (B) Body bends per minute of the indicated strains. WDR-23 rescue denotes *wdr-23* mutants expressing transgenes containing *wdr-23a* cDNA under control of the *wdr-23* promoter. Locomotion was quantified in one minute intervals in the absence of food (Student's *t-*test). (C) Percent time spent in locomotion of the indicated strains. *wdr-23a* cDNA was used for rescue with either the endogenous *wdr-23* promoter (WDR-23 rescue) or the *ges-1* promoter (intestinal rescue). The *skn-1* allele *zu67* was used for movement analysis. Animals were assayed for three minute intervals in the presence of food. (ANOVA, Tukey's post-hoc). (D) Rates of worm paralysis of the indicated strains when exposed to the acetylcholine esterase inhibitor aldicarb (1.0 mM). Wild type indicated by wt. WDR-23 rescue and over-expression (o/e) were completed using a genomic *wdr-23* fragment. WDR-23-GFP rescue denotes co-injection of *wdr-23a* and *wdr-23b* cDNA driven by the *wdr-23* promoter. (E) Rates of worm paralysis of the indicated strains when exposed to the cholinergic agonist levamisole (200 µM). WDR-23 rescue denotes *wdr-23* mutants expressing genomic *wdr-23* transgenes. (Error bars indicate ±SEM. *p<0.05, **p<0.01, ***p<0.001.)

We previously identified *wdr-23* in an RNA interference (RNAi) screen for genes that regulate synaptic transmission at the *C. elegans* neuromuscular junction [24]. To further characterize the effects of *wdr-23* function on the nervous system, we examined putative null *wdr-23(tm1817)* mutants, which have a 635 bp deletion, generating a premature stop in the third WD repeat (Figure 1A). In addition to their small size and delayed growth rates [19], *wdr-23* mutants had reduced locomotion rates and spent significantly less time moving compared to wild type animals (Figure S1A–S1C, Figure 1B and 1C, and Table S1). Transgenes expressing *wdr-23* cDNA driven by a 1.3 kb *wdr-23* promoter fragment fully rescued these phenotypes of *wdr-23* mutants, including the locomotion defects (Figure S1C, Figure 1B and 1C, and data not shown). Impaired locomotion could be due to presynaptic defects in neurotransmitter release or postsynaptic defects in muscle response to acetylcholine at NMJs. To distinguish between these possibilities, we examined the response of *wdr-23* mutants to drugs that alter neuromuscular function. Aldicarb is an acetylcholine esterase inhibitor, and treatment with aldicarb causes acetylcholine to accumulate in synaptic clefts, resulting in muscle contraction and eventual paralysis. Mutations that decrease the rate of acetylcholine release from motor neurons reduce the rate of aldicarb-induced paralysis, whereas mutations that increase acetylcholine release increase the rate of paralysis [25]. *wdr-23* mutants were highly resistant to the paralytic effects of aldicarb, and expression of a genomic *wdr-23* fragment in *wdr-23* mutants completely rescued this defect (Figure 1D). These results indicate that *wdr-23* mutants have impaired acetylcholine release from motor neurons. Consistent with these results, *wdr-23* mutants were hypersensitive to the paralytic effects of the muscle agonist levamisole (Figure 1E), indicating that muscle responsiveness was not reduced in these mutants.

### SKN-1 Mediates the Effects of *wdr-23* on Neuromuscular Function

**Figure 2:**
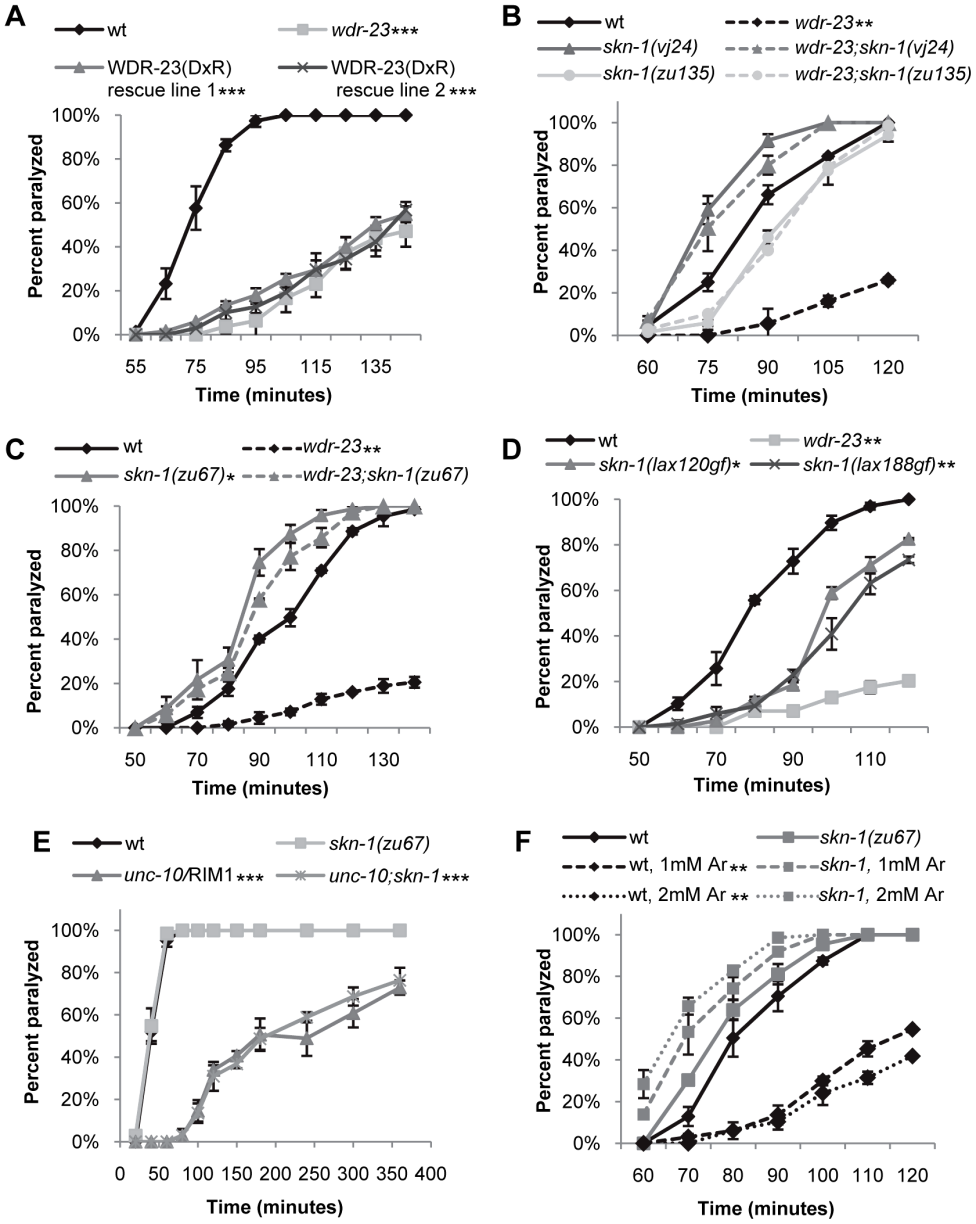
Loss of *skn-1* suppresses the aldicarb resistance of *wdr-23* mutants. Rates of worm paralysis of the indicated strains when exposed to aldicarb. (A) Expression of WDR-23(DxR) driven by the endogenous *wdr-23* promoter does not rescue the aldicarb phenotype of *wdr-23* mutants (1.0 mM aldicarb). (B) *skn-1(vj24)* encodes a R519STOP mutation, and *skn-1(zu135)* encodes a Q553STOP mutation. Both are in the DNA binding domain and are predicted to affect all three *skn-1* isoforms. (C) *skn-1(zu67)* encodes a R239STOP that specifically affects *skn-1a/c*. (D) *skn-1(gf)* alleles *lax188* and *lax120*, which encode E237K and S245L, respectively, are resistant to aldicarb induced paralysis. (E) Loss of *skn-1(zu67)* does not suppress the aldicarb resistance of *unc-10* mutants when exposed to 4.0 mM aldicarb. (F) Exposure to arsenite (Ar) results in *skn-1-*dependent resistance to aldicarb. The indicated strains were exposed to 0, 1.0, or 2.0 mM arsenite in NGM for 14 hours then allowed to recover for 3 hours prior to assay. (Error bars indicate ±SEM. *p<0.05, **p<0.01, ***p<0.001.)

Mammalian WDR23 binds to the DDB1-CUL4 ubiquitin ligase complex via an interaction between the cullin adaptor DDB1 and a conserved ‘DxR’ box within the WDR40 repeat domains [21], [22]. WDR-23 contains two conserved DxR boxes within WD repeats four and five (Figure 1A). To determine whether interaction with DDB1 is necessary for the function of WDR-23, we mutated the final arginines in the DxR boxes required for DDB binding to make WDR-23(DxR), which is predicted to be deficient in binding the DDB1 ortholog, DDB-1 [21], [22]. The WDR-23(DxR) mutation did not appear to alter the expression or localization of WDR-23 (Figure S2). However, expression of WDR-23(DxR) driven by the endogenous *wdr-23* promoter fragment failed to restore wild type aldicarb responsiveness to *wdr-23* mutants (Figure 2A). This suggests that the interaction between WDR-23 and DDB-1/CUL-4 is essential for regulating neuromuscular function.

To identify targets of WDR-23 that promote presynaptic function, we performed a forward genetic screen for suppressors of the *wdr-23* aldicarb resistant phenotype. We identified an allele of *skn-1*, *vj24*, which encodes a nonsense mutation in the DNA binding domain of SKN-1 (R519STOP of the *skn-1a* isoform); *skn-1(vj24)* completely restored the small size, delayed growth and aldicarb resistance of *wdr-23* mutants to wild type levels (Figure 2B and data not shown). An independently isolated *skn-1* allele, *zu135*, which also encodes a premature stop in the SKN-1 DNA binding domain, also completely restored the size, delayed growth and aldicarb response of *wdr-23* mutants (Figure 2B and data not shown). SKN-1 encodes three isoforms that share the DNA binding domain (www.wormbase.org). Two SKN-1 isoforms, SKN-1a and SKN-1c, function in the intestine to mediate stress response [4], [26], whereas the third isoform, SKN-1b, functions in two sensory neurons for dietary restriction-induced longevity [13]. *skn-1(zu67)* mutants, which specifically lack the *skn-1a* and *skn-1c* isoforms, restored wild type locomotion rates, aldicarb response, size and growth rates to *wdr-23* mutants (Figure 1C, Figure 2C, Figure S1B and S1C). None of the *skn-1* mutants examined displayed significant size, growth, locomotion or aldicarb response defects alone (Figure 2B and 2C, Figure S1B, and data not shown). These results suggest that the primary function of WDR-23 is to negatively regulate SKN-1a and/or SKN-1c, and increased SKN-1a/c activity leads to defects in synaptic function. Consistent with this idea, *skn-1* gain-of-function (gf) mutants, which are predicted to enhance the activity of SKN-1a and SKN-1c isoforms [27], displayed aldicarb resistance reminiscent of *wdr-23* mutants (Figure 2D). Thus, *wdr-23* is likely to promote neuromuscular function by negatively regulating SKN-1 activity.

Because SKN-1 induces transcriptional programs that detoxify xenobiotics, the aldicarb resistance of *wdr-23* mutants could be due to increased aldicarb detoxification by SKN-1 in these mutants rather than a specific effect of SKN-1 activity on synaptic function. If this were the case, we would predict that the aldicarb resistance of mutants that directly reduce acetylcholine secretion should also be suppressed by *skn-1* mutations. *unc-10/*RIM1 is a presynaptic protein that is required for acetylcholine release from synaptic vesicles, and *unc-10* mutants are aldicarb resistant [25]. However, *skn-1(zu67)* mutations failed to suppress the aldicarb resistant phenotype of *unc-10/*RIM1 mutants (Figure 2E). Thus, the aldicarb and locomotion defects observed in *wdr-23* mutants likely reflect SKN-1-dependent changes in neuromuscular function, rather than alterations in drug detoxification.

### Environmental Stress Alters NMJ Function

Several environmental stressors have been identified that promote SKN-1 nuclear translocation, induce SKN-1-dependent transcriptional programs, and have SKN-1-dependent effects on stress response and aging [4], [19], [28], [29]. In order to identify stressors that might cause SKN-1-dependent changes in neuronal function, we tested a variety of conditions that activate SKN-1 for their effects on aldicarb-induced paralysis. Interestingly, treatment of wild type animals with paraquat, peroxide or heat shock did not cause aldicarb resistance in our assays (data not shown). In contrast, we found that prolonged exposure to the toxin sodium arsenite resulted in robust aldicarb resistance (Figure 2F). Arsenite stimulates reactive oxygen species production and protein misfolding and has previously been shown to cause SKN-1 nuclear translocation in intestinal cells [7], [30]. Importantly, arsenite-induced aldicarb resistance was completely blocked by *skn-1* mutations (Figure 2F). In contrast, no change in aldicarb responsiveness was observed following acute exposure to arsenite, consistent with previous findings (data not shown, [31]). Together, these results suggest arsenite may reduce NMJ function by inducing SKN-1 dependent transcriptional programs.

### *wdr-23* Mutants Have Defective Neurotransmitter and Neuropeptide Release

**Figure 3:**
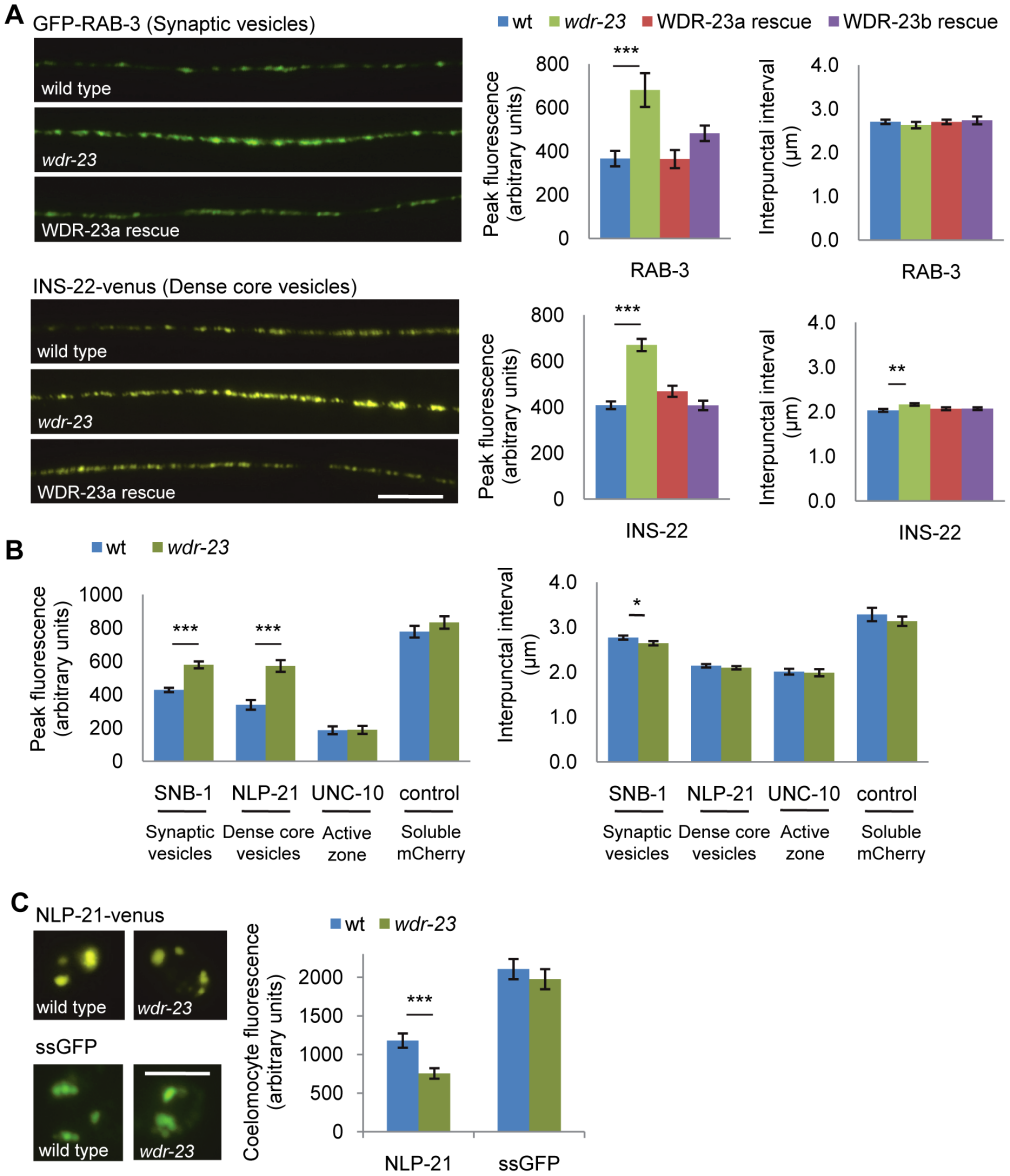
Synaptic vesicle and dense core vesicle secretion defects of *wdr-23* mutants. (A) *Left*, Representative images of the distribution of the synaptic vesicle protein GFP-RAB-3 and the pro-neuropeptide INS-22-YFP driven by the *unc-17*/VAChT promoter in cholinergic motor neurons in young adult wild type, *wdr-23* mutants and rescued *wdr-23* mutants expressing WDR-23a cDNA driven by the endogenous *wdr-23* promoter and integrated via Mos insertion. *Right*, Quantification of the peak fluorescence and the interpunctal interval (Student's *t-*test). (B) Quantification of the peak fluorescence (*left*) and interpunctal interval (*right*) of the synaptic vesicle associated protein GFP-SNB-1/synaptobrevin, the pro-neuropeptide protein NLP-21-YFP, and the active zone protein UNC-10/RIM1-GFP. Control refers to soluble mCherry expressed under the *unc-17/*VAChT promoter. (C) *Left*, Representative images of coelomocyte fluorescence in wild type and *wdr-23* L4 stage animals expressing NLP-21-YFP. *Right*, Quantification of NLP-21-YFP in coelomocytes of wild type and *wdr-23* mutants. ssGFP refers to GFP tagged to signal sequence which results in a constitutively secreted version of GFP (Student's *t-*test). (Scale bar represents 10 µm; Error bars indicate ±SEM. *p<0.05, **p<0.01, ***p<0.001.)

**Figure 4:**
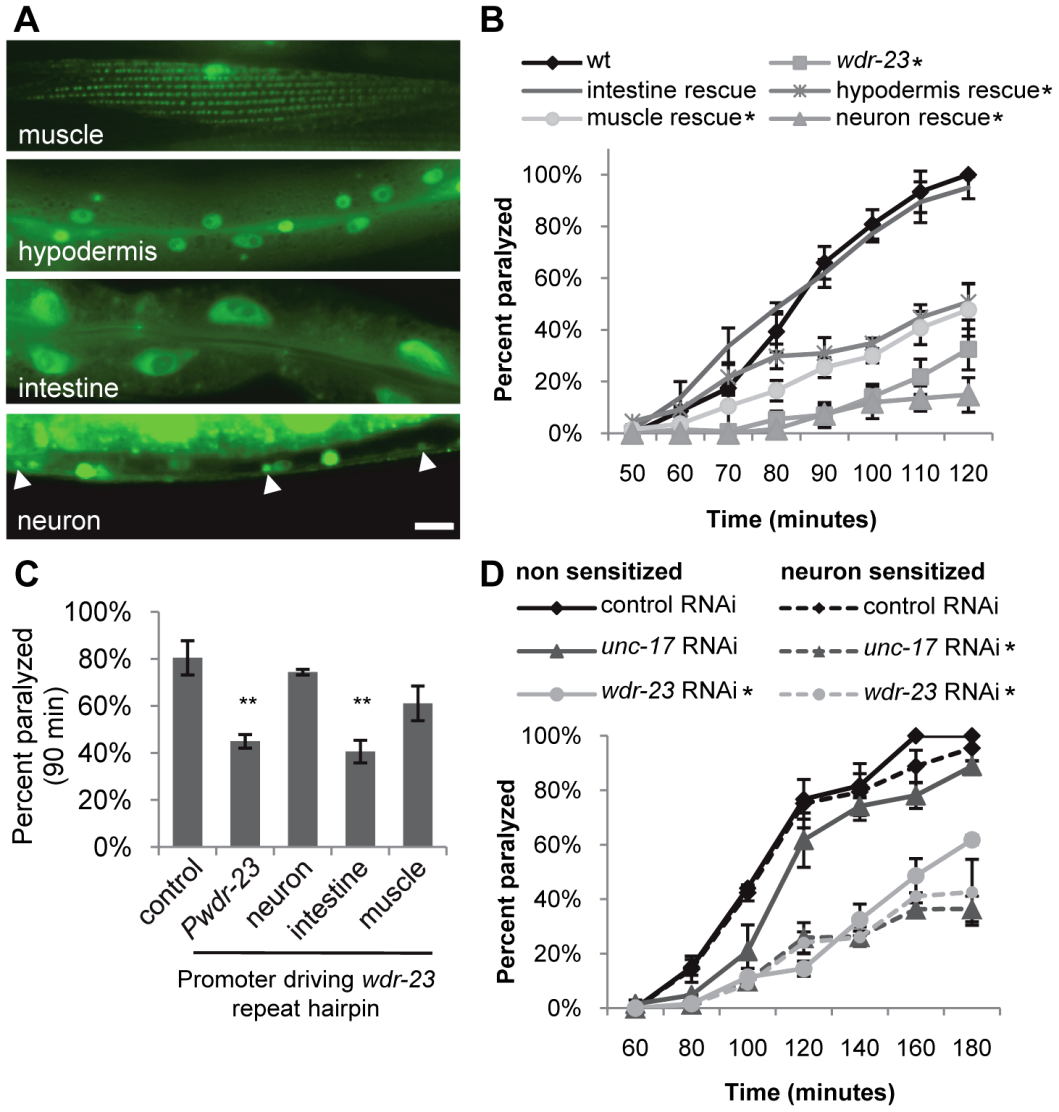
WDR-23 expression pattern and site of action. (A) Representative images showing fluorescence in the indicated tissues from transgenic animals expressing nuclear-localized *gfp* from the endogenous 1.3 kb *wdr-23a* promoter fragment. Arrowheads indicate neuronal cell bodies. (B–D) Paralysis on 1.0 mM aldicarb of the indicated strains. For rescue and knockdown, the following promoters were used: *ges-1* (intestine), *rab-3* (neuron), *col-12* (hypodermis), and *myo-3* (muscle). Intestinal and neuronal rescues were independently verified using *nlp-40* and *snb-1* promoters, respectively (data not shown). (B) Rates of paralysis of tissue-specific rescue of *wdr-23* mutants. (C) Hairpin RNAi knockdown of *wdr-23* in a *sid-1(qt2)* background results in tissue-specific aldicarb resistance after 90 minutes on aldicarb (Student's *t-*test). *wdr-23* indicates knockdown with endogenous *wdr-23a* promoter. (D) RNAi knockdown by feeding of the indicated genes in a wild type or neuronally sensitized (*nre-1 lin-15b*) strain. Control is the empty expression vector L4440. (Scale bar represents 10 µm; Error bars indicate ±SEM. *p<0.05, **p<0.01.)

We next examined the nature of the neuronal defects in *wdr-23* mutants in more detail by analyzing synaptic structure and function. Examination of cholinergic neurons labeled with GFP (using the *acr-2* promoter [32]) revealed no gross morphological changes in axonal projections or in the number of cholinergic neurons in *wdr-23* mutants (wild type = 31.0±0.6; *wdr-23* = 31.9±0.4, *p* = 0.2 Student's *t-*test, and Figure S1A). In addition, quantification of the active zone protein UNC-10/RIM1 tagged to GFP [33] showed no significant changes in the number of synapses (measured by interpunctal interval, Figure 3B and Table S2) or synaptic size (measured by punctal width, wild type = 0.75±0.03 µm; *wdr-23* = 0.72±0.02 µm, *p* = 0.3) in *wdr-23* mutants compared to wild type controls. These results indicate that *wdr-23* mutants are likely to have normal neuronal and synaptic development. In support of this idea, we found that RNAi knockdown of *wdr-23* after hatching (when development is largely complete) resulted in aldicarb resistance similar to that seen in *wdr-23* mutants (Figure 4D).

To investigate defects in neurotransmitter release, we visualized the cycling of synaptic vesicles at cholinergic synapses *in vivo*. Synaptic vesicle-associated proteins labeled with GFP can be used to assess synaptic vesicle cycling [33], [34]; in axons, the fluorescent intensity of individual puncta labeled with synaptic vesicle markers correlates with the number of vesicles at presynaptic sites [34]. Along these lines, we utilized two synaptic vesicle markers, GFP-SNB-1 and GFP-RAB-3, to visualize steady-state vesicle abundance at release sites in *wdr-23* mutants. We found that mutants lacking *wdr-23* had significantly increased GFP-SNB-1 and GFP-RAB-3 punctal fluorescence, but not axonal fluorescence, relative to wild type controls. The increase in punctal fluorescence of GFP-RAB-3 in *wdr-23* mutants was fully rescued by WDR-23 cDNA (Figure 3A, 3B and Table S2). These results are consistent with a defect in synaptic vesicle exocytosis from cholinergic motor neurons in *wdr-23* mutants.

*C. elegans* cholinergic axons also secrete the neuropeptides NLP-21 and INS-22, which are FMRF amide-related peptide (FaRP) and an insulin-like growth factor, respectively, and are packaged in dense core vesicles [35]. Fluorescently tagged versions of these proteins adopt a punctate pattern in axons of cholinergic motor neurons, and defects in dense core vesicle secretion release result in increased punctal fluorescence within axons. We found that *wdr-23* mutants had significant increases in INS-22-YFP and NLP-21-YFP punctal fluorescence, but no change in axonal fluorescence, compared to wild type controls (Figure 3A, 3B and Table S2), consistent with a defect in dense core vesicle secretion. Expression of WDR-23 cDNA fully rescued the increases in punctal fluorescence of INS-22-YFP in *wdr-23* mutants to wild type levels. Next, we examined neuropeptide accumulation in specialized scavenger cells called coelomocytes which endocytose neuropeptides after they are secreted from neurons [36]. Mutants with defective neuropeptide secretion accumulate less fluorescence in coelomocytes [35]. We found that coelomocyte fluorescence in *wdr-23* mutants secreting NLP-21-YFP from motor neurons was significantly dimmer than in wild type controls (Figure 3C and Table S2). In contrast, coelomocyte fluorescence in *wdr-23* mutants secreting GFP fused to a signal sequence (ssGFP) from motor neurons [37] was not significantly different than wild type controls, indicating that constitutive vesicle release and coelomocyte uptake are normal in *wdr-23* mutants (Figure 3C and Table S2). Together, our results suggest that *wdr-23* specifically promotes the regulated exocytosis of synaptic vesicles and dense core vesicles from motor neurons.

### WDR-23 Functions in the Intestine to Regulate Synaptic Transmission

**Figure 7:**
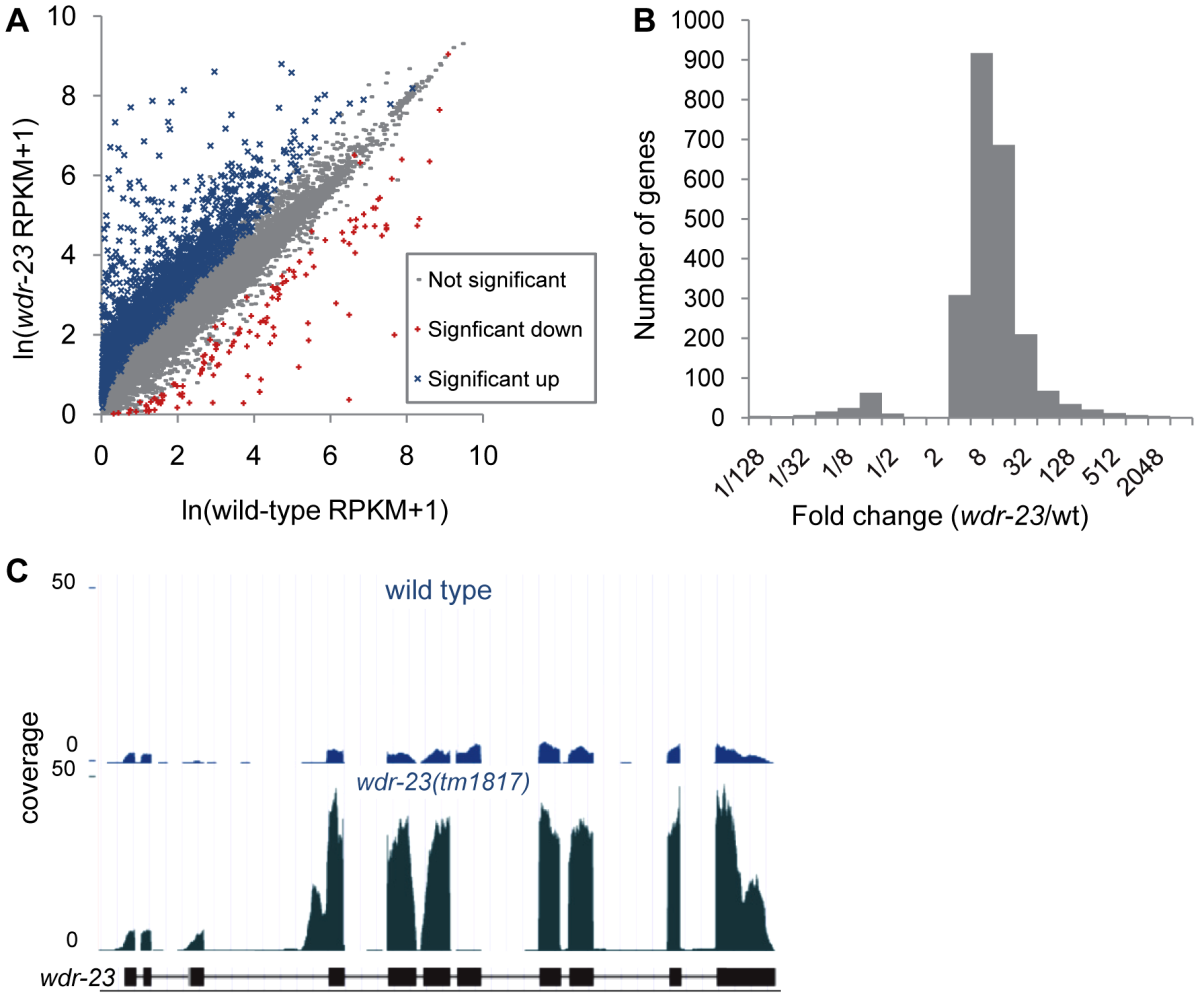
Analysis of whole-transcriptome RNA sequencing of *wdr-23* mutants. (A) Correlation plot of RNA-seq reads for wild type versus *wdr-23* mutants. Genes significantly different are indicated in blue (up-regulated) and red (down-regulated). (B) Distribution of fold change of genes significantly different in *wdr-23* mutants relative to wild type controls. (C) Coverage plot of RNA-seq reads of *wdr-23* in wild type and *wdr-23(tm1817)* mutants.

To determine in which tissues WDR-23 functions to regulate neurotransmitter release, we first examined the expression pattern of *wdr-23*. *wdr-23* encodes two isoforms, WDR-23a and WDR-23b, distinguishable by their unique amino termini (Figure 1A; www.wormbase.org). *wdr-23a* is the predominant isoform (Figure 7C), and the endogenous 1.3 kb *wdr-23a* promoter fragment driving *wdr-23a* fully rescued *wdr-23* locomotion phenotypes (Figure 1B and 1C). A reporter consisting of the 1.3 kb *wdr-23a* promoter tagged to *gfp* was detected in the hypodermis (skin), intestine, muscle and neurons, consistent with previous findings (Figure 4A, [19]). A 2.0 kb *wdr-23b* promoter fragment tagged to *gfp* was detected in muscle, hypodermis, and intestine, but not in neurons (Figure S3). Next, we selectively expressed *wdr-23* in different tissues using tissue-specific promoters and assayed its ability to rescue the neuromuscular defects of *wdr-23* mutants. Expression of functional WDR-23-GFP in the intestine (using either the *ges-1* or *nlp-40* promoter) fully restored wild type aldicarb responses and locomotion to *wdr-23* mutants (Figure 1C, Figure 4B and data not shown). In contrast, expression of WDR-23-GFP in muscle (*myo-3* promoter) or neurons (*rab-3* or *snb-1* promoter) was not sufficient to restore normal aldicarb responsiveness to *wdr-23* mutants (Figure 4B).

To confirm the intestinal site of action of *wdr-23*, we knocked down *wdr-23* by tissue-specific RNA interference. Neurons of wild type animals are resistant to gene knockdown by feeding double stranded RNA (dsRNA), whereas neurons of *nre-1 lin-15b* mutants are less resistant [38], [39]. *unc-17* encodes the vesicular acetylcholine transporter that functions specifically in neurons, and *unc-17/*VAChT loss-of-function mutants are aldicarb resistant [25]. As expected, feeding knockdown of *unc-17/*VAChT was only effective in eliciting aldicarb resistance in *nre-1 lin-15b* mutants, but not in wild type animals (Figure 4D), confirming that *unc-17/*VACht acts in neurons. In contrast, *wdr-23* knockdown elicited robust aldicarb resistance in both wild type animals and *nre-1 lin-15b* mutants (Figure 4D), suggesting that *wdr-23* activity is likely to be required outside of the nervous system for its effects on aldicarb response. Next, we selectively knocked down *wdr-23* activity in specific tissues by expressing inverted repeat hairpin transgenes directed toward *wdr-23* using tissue specific promoters (in a *sid-1* mutant background to prevent systemic spreading of RNAi) [40], [41]. *wdr-23* hairpin expression in the intestine (using *ges-1* and *nlp-40* promoters) but not in neurons or muscles (using the *rab-3* and *myo-3* promoters, respectively) resulted in aldicarb resistance comparable to that seen with the endogenous *wdr-23* promoter fragment (Figure 4C and Table S1). Together, these results indicate that *wdr-23* in the intestine is necessary and sufficient to positively regulate synaptic function and suggest that activation of SKN-1 in the intestine is responsible for the reduced neuronal function of *wdr-23* mutants.

### WDR-23 Localization and Function

**Figure 5:**
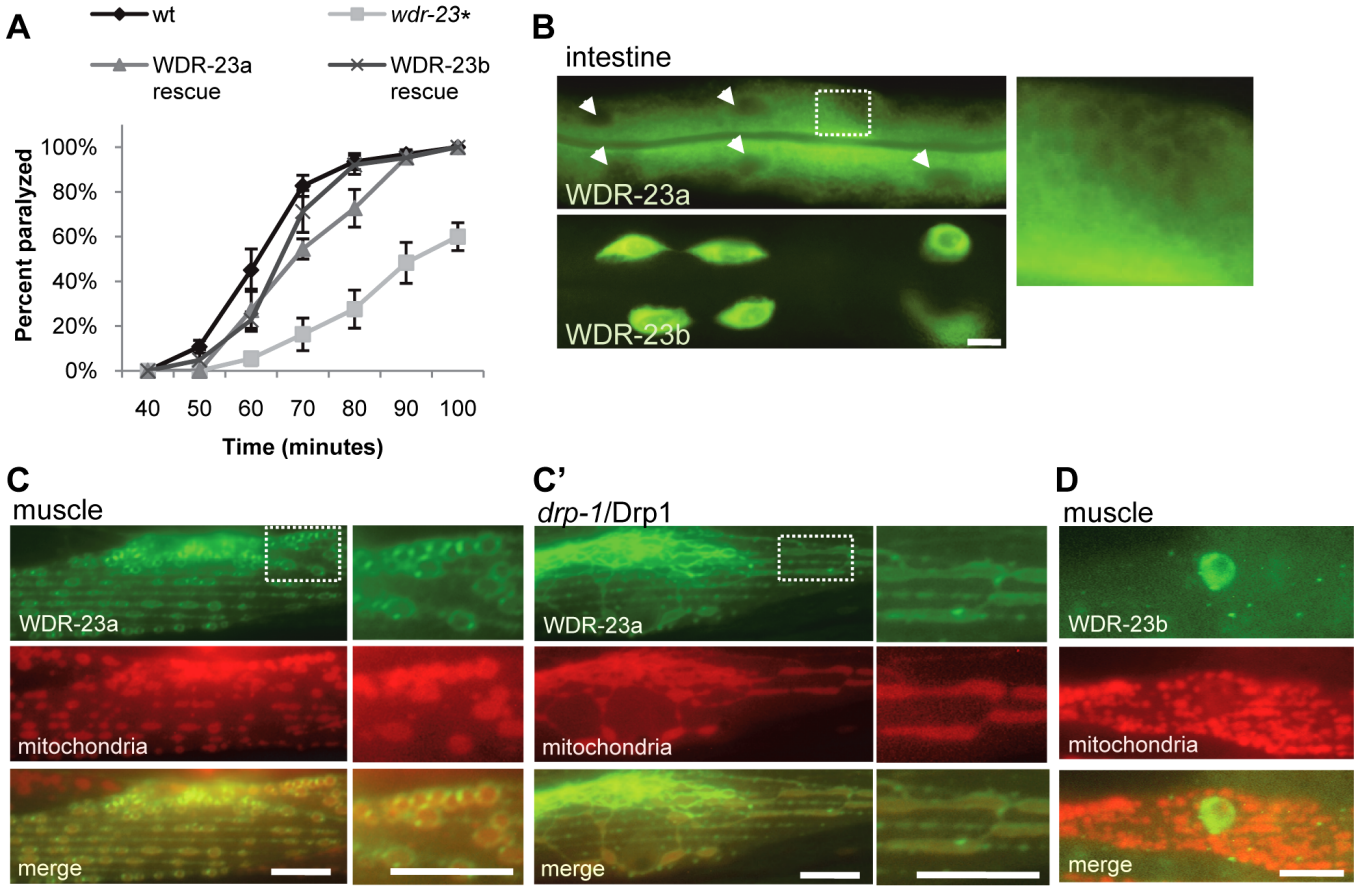
Subcellular localization and function of WDR-23 isoforms. (A) Rates of paralysis of the indicated strains on 1.0 mM aldicarb. WDR-23a and WDR-23b rescues denote *wdr-23* mutants expressing single copy transgenes containing *wdr-23a* or *wdr-23b* cDNA, respectively, driven by the endogenous *wdr-23* promoter generated by Mos insertion. (B) Intestinal expression of WDR-23a-GFP and WDR-23b-GFP driven by the *ges-1* promoter in a *glo-1* background to reduce autofluorescence. Box highlights ring-like pattern of WDR-23a-GFP localization. Arrowheads indicate intestinal nuclei. (C) WDR-23a-GFP co-localization with the inverted outer mitochondrial membrane marker (INVOM-RFP) in muscle cells in wild type and *drp-1* mutants. (D) WDR-23b-GFP localization in muscle cells; mitochondria are labeled with INVOM-RFP for reference (Scale bar represents 10 µm; Error bars indicate ±SEM. *p<0.05.)

Next, to determine the sub-cellular localization of WDR-23, we expressed functional GFP tagged versions of WDR-23a or WDR-23b in the intestine, and we found that the two isoforms localized to distinct sub-cellular compartments. WDR-23b-GFP was entirely nuclear, whereas WDR-23a-GFP adopted a ring-like pattern of fluorescence in the intestinal cytoplasm (Figure 5B). The identity of the ring-like structures could not be determined unambiguously by co-localization studies, since fluorescent markers were difficult to resolve in the intestine (Figure S4). Thus, we examined WDR-23a-GFP localization in muscles—which also express *wdr-23* but are more amenable to fluorescent imaging studies [42].

In muscles, WDR-23a-GFP fluorescence was observed at dense bodies, which are actin rich-sites of muscle attachment to the hypodermis, and on ring-like structures similar to those seen in the intestine (Figure 5C). The ring-like structures were regularly spaced along muscle fibers and co-localized with an outer mitochondrial membrane marker tagged with RFP (INVOM-RFP). WDR-23a-GFP fluorescence appeared to be distributed on the outer surface of the rings occupied by the mitochondrial marker, suggesting that it may be associated with outer mitochondrial membranes (Figure 5C). To test this idea, we examined the distribution of WDR-23a-GFP in *drp-1/*Drp1 loss of function mutants. Drp1 is required for outer mitochondria membrane fission; *C. elegans* mutants lacking *drp-1* activity have normal inner membrane fission, but form continuous elongated tubes of outer membrane [43]. The WDR-23a-GFP fluorescence pattern was dramatically altered in *drp-1* mutants; fluorescence was seen in elongated tubes that co-localized with INVOM-RFP, consistent with WDR-23a-GFP being associated with the outer mitochondrial membrane (Figure 5C′). In contrast, WDR-23b-GFP was detected exclusively in nuclei (Figure 5D).

**Figure 6:**
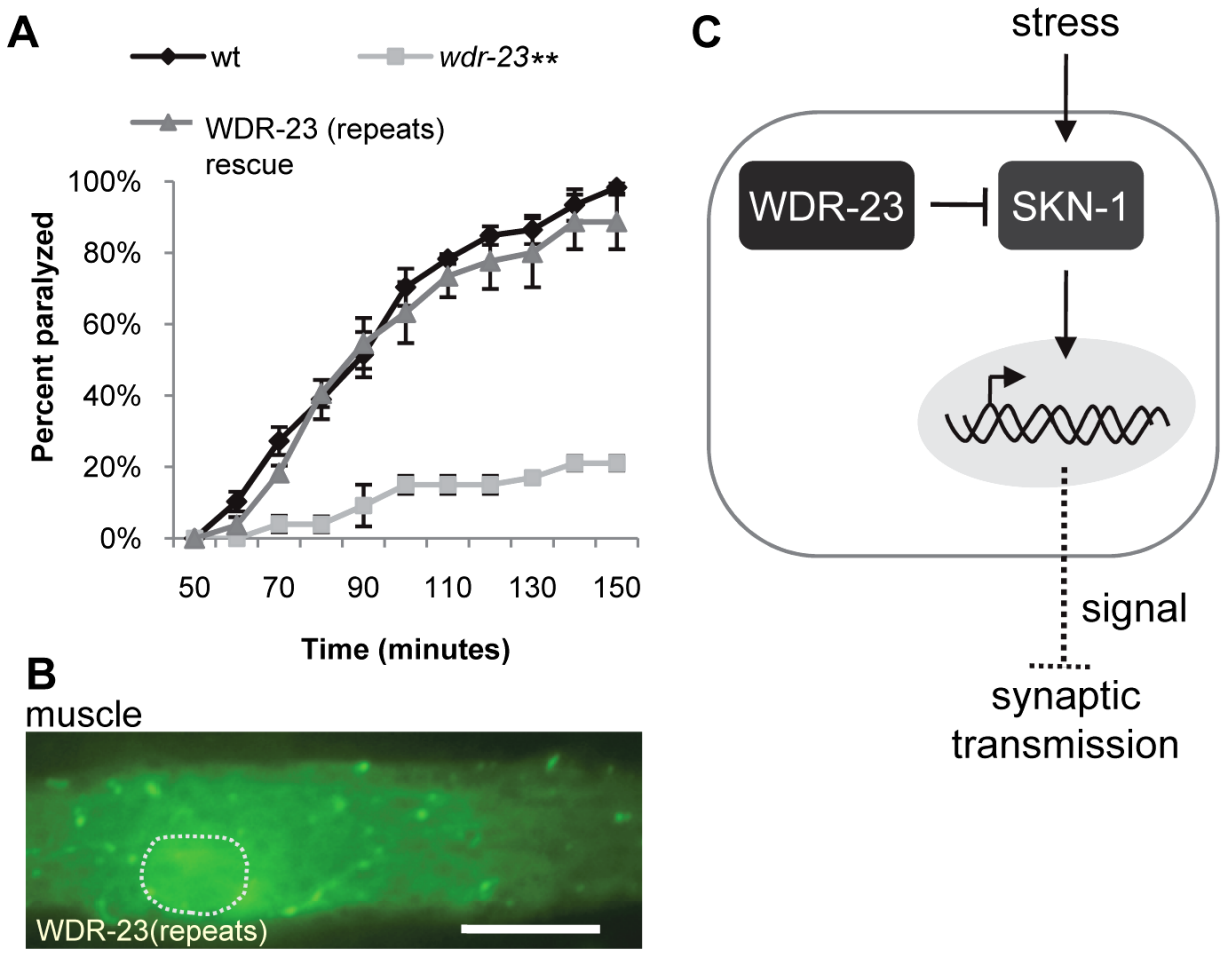
The WD40 repeat domains are sufficient for WDR-23 function. (A) Rates of worm paralysis of the indicated strains when exposed to 1.0 mM aldicarb. WDR-23(repeats) rescue denotes *wdr-23* mutants expressing transgenes carrying a *wdr-23* variant that only contains the seven WD40 repeats driven by the endogenous *wdr-23* promoter. (B) WDR-23(repeat) tagged to GFP and expressed in muscle. Dotted circle denotes nucleus. (Scale bar represents 10 µm; Error bars indicate ±SEM. **p<0.01.) (C) Model for SKN-1 regulation of synaptic function. In this model, WDR-23 inhibits SKN-1. Activation of SKN-1 in the intestine, either genetically through loss of *wdr-23* or through environmental stress such as arsenite, increases transcription of target genes and may promote the release of a diffusible factor or factors. Endocrine signaling reduces synaptic vesicle and dense core vesicle release, resulting in decreased neurotransmission at neuromuscular junctions.

To test whether mitochondrial or nuclear localization of WDR-23 is critical for its function, we introduced *wdr-23* isoforms into *wdr-23* mutants using Mos-mediated low copy insertion [44] to avoid artifacts due to high copy expression of *wdr-23*. We found that both WDR-23a and WDR-23b transgenes restored normal aldicarb responsiveness, synaptic vesicle and dense core vesicle release to *wdr-23* mutants (Figure 3A and Figure 5A). Last, a truncated version of WDR-23 that contains only the common WD40 repeat region, referred to as WDR-23(repeats), also fully restored aldicarb response of *wdr-23* mutants to wild type (Figure 6A and 6B). This variant was not concentrated at mitochondria or nuclei but was distributed uniformly throughout the muscle. Taken together, these results indicate that the functional domain of WDR-23 is contained within the WD40 repeats, and although WDR-23 isoforms are concentrated at distinct cellular compartments, the precise sub-cellular localization of WDR-23 may not be critical for its function.

### Transcriptional Targets of SKN-1 Are Increased in *wdr-23* Mutants

What might be the critical transcriptional targets of SKN-1 that inhibit synaptic transmission? To begin to address this question, we compared gene expression profiles of *wdr-23* mutants to wild type controls using whole transcriptome RNA sequencing. Approximately 29.2 and 29.4 million reads were mapped to the genome for wild type and *wdr-23* mutants, respectively, corresponding to an average coverage of 698.9 reads per gene for wild type and 728.5 reads per gene for *wdr-23* mutants. Using the Cuffdiff module of the statistical Cufflinks package [45], we identified 2285 genes that were significantly up-regulated and 134 genes that were significantly down-regulated in *wdr-23* mutants (Figure 7A, Tables S4 and S6). The amplitude of the up-regulated genes ranged from 1.04 (*rpl-3/*ribosomal protein L3, *p* = 0.001) to 3517.69 fold (*clec-74/*c-type lectin, *p*<0.001), with an average fold change of 22.9 (Figure 7B). Significantly down-regulated genes ranged from 0.001 (*ocr-1/*capsaicin receptor related, *p*<0.001) to 0.96 fold (*eft-3/*elongation factor 1-alpha, *p*<0.001) and had a mean fold change of 0.16 (Figure 7B). Microarray analysis has previously identified 118 genes that are induced by arsenite in a SKN-1 dependent manner [7], [17]. We identified 80 genes among these that were significantly up-regulated in *wdr-23* mutants (Table S4), supporting the idea that SKN-1-dependent transcriptional programs are increased in *wdr-23* mutants.

**Figure 8:**
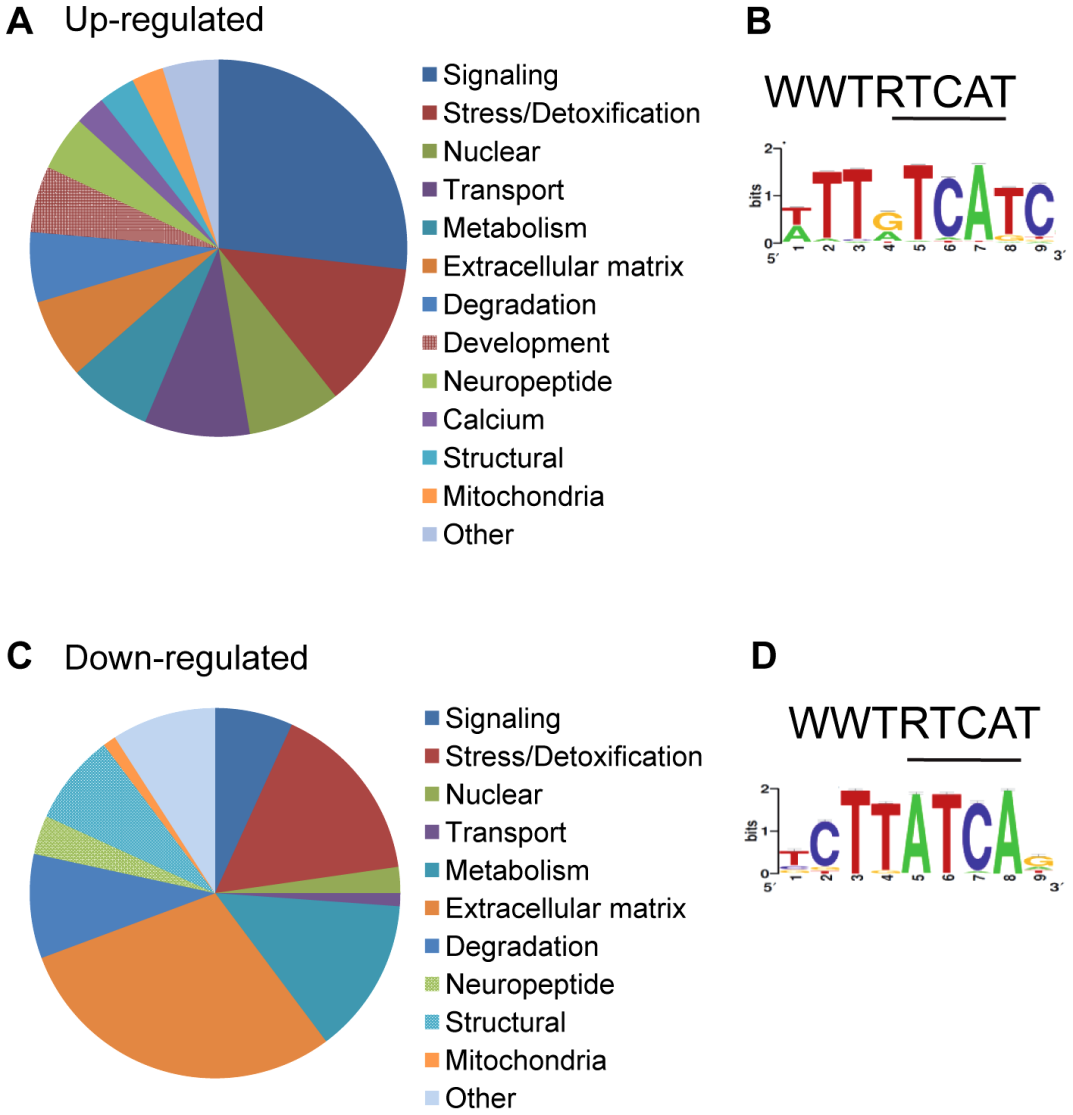
Functional analysis of genes significantly different in *wdr-23* mutants. (A–B) Analysis of 2285 up-regulated genes in *wdr-23* mutants. (A) Representation of functional groups of up-regulated genes with known function (1316 genes) in *wdr-23* mutants relative to wild type classified by GO terms and protein domain established by DAVID analysis. (B) Canonical SKN-1 binding sequence and enrichment of SKN-1 binding motif identified by RSAT oligonucleotide analysis. One kilobase of intergenic sequence upstream of each ORF for all 2285 up-regulated genes was used for RSAT analysis. RSAT consensus represented by WebLogo. (C–D) Analysis of 134 genes down-regulated in *wdr-23* mutants. (C) Representation of functional groups of down-regulated genes with known function (88) in *wdr-23* mutants relative to wild type. (D) Canonical SKN-1 binding sequence and enrichment of SKN-1 binding motif identified by RSAT oligo analysis.

We classified all significantly up-regulated genes in *wdr-23* mutants using the Database for Annotation, Visualization and Integrated Discovery (DAVID) by GO terms and InterPro protein domains (Figure 8A and Table 1) [46]. Approximately twenty percent of genes with known functions were classified as being involved in metabolism or stress resistance and detoxification. Among these are genes involved in SKN-1/Nrf2 dependent oxidative and xenobiotic stress resistance, including glutathione *S-*transferases, UDP-glucuronosyl transferases and cytochrome P450s [7], [17]. Two glutathione *S-*transferases, *gst-4* and *gst-30*, whose expression is increased by SKN-1 activation [7], [47], increased by 255.5 and 1921.1 fold, respectively in *wdr-23* mutants. We validated these increases and confirmed that their expression was SKN-1 dependent by qRT-PCR (Table 2).

**Table 1:** Functional classification of genes up-regulated in *wdr-23* mutants.

Category	GO Term	Count	*p* value	Enrichment
Biological Process	metabolic process	484	0.000	16.924
	neuropeptide signaling pathway	26	0.000	7.061
	G-protein coupled receptor protein signaling pathway	62	0.000	6.860
	cellular lipid metabolic process	29	0.000	2.405
	cellular amino acid derivative metabolic process	11	0.001	2.027
	regulation of feeding behavior	9	0.016	1.762
	locomotory behavior	14	0.001	1.586
	mating	6	0.012	1.588
	ion transport	69	0.008	1.284
	learning	5	0.041	1.223
	dauer larval development	15	0.040	1.123
	synaptic transmission	14	0.012	1.043
	hyperosmotic response	5	0.052	0.984
	chemical homeostasis	10	0.009	0.980
	amine transport	8	0.023	0.858
	response to oxidative stress	8	0.029	0.747
Cellular Compartment	cytoskeleton	40	0.000	2.223
	myosin complex	6	0.007	1.794
	synapse	19	0.009	1.460
	intrinsic to plasma membrane	13	0.023	1.255
	microtubule cytoskeleton	10	0.021	1.206
	mitochondrial membrane	7	0.052	0.380
Molecular Function	hydrolase activity	231	0.000	21.528
	transferase activity	183	0.000	9.460
	kinase activity	121	0.000	9.142
	iron ion binding	60	0.000	2.946
	lipase activity	17	0.000	2.574
	cation binding	248	0.005	1.554
	peptidase activity	54	0.041	1.335
	phospholipase activity	9	0.016	1.304
	antioxidant activity	11	0.006	1.287
	transmembrane transporter activity	89	0.004	1.276
	muscarinic acetylcholine receptor activity	3	0.033	1.207
	serine-type peptidase activity	14	0.067	0.903
	2 iron, 2 sulfur cluster binding	5	0.045	0.744
	active transmembrane transporter activity	32	0.014	0.606
	neurotransmitter:sodium symporter activity	5	0.049	0.578
	Ras guanyl-nucleotide exchange factor activity	6	0.041	0.485

**Table 2:** qRT–PCR validation of RNA–seq.

	qRT-PCR			RNAseq
Gene	*wdr-23 ± SEM*	*skn-1 ± SEM*	*skn-1;wdr-23 ± SEM*	*wdr-23*
*gst-4*	**31.31±10.69**	0.59±0.56	1.29±0.36	**255.98**
*gst-30*	**580.83±151.96**	**5.12±1.52**	**6.06±1.46**	**1920.69**
*cdc-42*	0.91±0.17	0.86±0.20	0.85±0.19	1.11
*pmp-3*	1.03±0.04	1.61±0.24	1.02±0.13	0.95
*snt-1*	**3.42±1.38**			**3.41**
*unc-25*	**3.23±0.53**			**2.80**
*unc-17*	1.28±0.33			1.04

Legend: qRT-PCR values have been normalized to wild type. Significant changes are indicated in bold. *skn-1(zu67)* was used for all qRT-PCR experiments. *rpl-2* was used for normalization. *cdc-42* and *pmp-3* were used as negative controls.

In addition to stress response and metabolic genes, several other categories of genes were up-regulated in *wdr-23* mutants, including those involved in signaling (27%), protein degradation (6%) and the extracellular matrix (7%). In addition, 165 genes (13%) likely involved in neuronal function or behavior were up-regulated (Table S5). Genes directly involved in synaptic transmission and calcium sensing at neuromuscular junctions, such as *unc-25/*glutamic acid decarboxylase, *snt-1/*synaptotagmin, *unc-13/*Munc13, and *unc-10/*RIM1, all had statistically increased expression in *wdr-23* mutants (Table S5). qRT-PCR of *unc-25/*glutamic acid decarboxylase and *snt-1/*synaptotagmin (but not the negative control *unc-17/*VAChT) verified a roughly three-fold increase in expression in *wdr-23* mutants, in agreement with the RNA sequencing results (Table 2). Finally, approximately 50 neuropeptide genes expressed in diverse tissues were up-regulated in *wdr-23* mutants, suggesting that endocrine signaling is extensively altered in these mutants.

To determine the number of up-regulated genes in *wdr-23* mutants likely to be direct binding targets of SKN-1, we used Regulatory Sequence Analysis Tools (RSAT) oligo analysis [48] to detect and quantify potential SKN-1 binding motifs. Previous studies have established the SKN-1 consensus to be WWTDTCAT or the related sequence TTDTCATC (W = A/T, D = G/A/T), where the underlined sequence is thought to be directly bound by SKN-1 [4], [7], [49]. Consistent with these studies, we found the sequence WWTDTCAT to be enriched in the promoter regions within 1 kb upstream of the transcriptional start of the most up-regulated genes. This motif was found in 90.3% of the top 300 up-regulated genes, compared to 78.6% of promoters from a randomized list of genes not significantly different in *wdr-23* mutants relative to wild type controls (Table S3). Further analysis by RSAT identified the more restrictive sequence TTDTCATC to be over-represented among these promoter fragments (Figure 8B). This restricted element was enriched in the stress related genes (found in 52% of the stress and detoxification gene promoters, compared to 25% of the randomized gene list; Table S3), consistent with these genes being direct SKN-1 targets. In contrast, this restrictive motif was found in only 24.6% of the promoters of the synaptic or behavioral genes, implying that a majority of these genes may not be direct SKN-1 targets. Thus, changes in gene expression are likely to reflect both direct and indirect regulation by SKN-1. Together, these results indicate that extensive changes in gene expression profiles of functionally diverse genes in multiple tissues takes place in *wdr-23* mutants.

*wdr-23(tm1817)* mutants have a deletion that spans exon seven (of *wdr-23a*), and examination of *wdr-23(tm1817)* mutants confirmed the presence of a truncated *wdr-23* transcript in which exons six and eight were spliced together (Figure 7C), resulting in a frame shift and premature stop. Surprisingly, *wdr-23* transcripts were 7.6 fold more abundant in *wdr-23* mutants compared to wild type controls (Figure 7C). The *wdr-23a* promoter contains three restrictive and five canonical SKN-1 binding sites within 1 kb upstream of the start site. Together, these results indicate that *wdr-23* expression itself may be under direct control of SKN-1 (Table S4).

Finally, using DAVID, we analyzed the functional classifications of the 134 genes down-regulated in *wdr-23* mutants (Figure 8C and Table 3). Grouping by GO terms revealed that genes involved in aging were enriched this group. Interestingly, previous microarray studies identified 63 genes to be transcriptionally down-regulated under basal conditions by SKN-1 but did not detect any genes to be down-regulated in response to stress [7]. RSAT promoter analysis identified the motif TCTTATCAG to be over-represented in the genes down-regulated in *wdr-23* mutants (Figure 8D). This sequence is different than the sequence TGAGTCAC, which is enriched in genes down-regulated by *skn-1* RNAi [7]. This suggests that genes constitutively down-regulated by SKN-1 might utilize a different binding site than genes down-regulated by SKN-1 during stress.

**Table 3:** Functional classification of genes down-regulated in *wdr-23* mutants.

Category	GO Term	Count	*p* value	Enrichment
Biological Process	aging	7	0.004	2.414
	lipid transport	5	0.000	1.760
	carbohydrate metabolic process	5	0.017	0.927
	defense response	3	0.049	0.541
Cellular Compartment	integral to membrane	38	0.027	0.931
Molecular Function	lipid transporter activity	5	0.000	3.379
	lysozyme activity	3	0.001	1.550
	cysteine-type peptidase activity	5	0.005	1.366

## Discussion

Inducible transcriptional programs are critical cellular mechanisms which allow organisms to respond systemically to varying environmental conditions such as caloric restriction, hypoxia or inflammation in order to promote survival. The conserved transcription factor SKN-1/Nrf2 has well known roles in preventing cellular damage caused by oxidative and xenobiotic stress. In this study, we establish that WDR-23 is the principle negative regulator of SKN-1 *in vivo*. We show that SKN-1/Nrf2 negatively regulates function of the *C. elegans* NMJ by reducing neurotransmitter secretion. We find that SKN-1/Nrf2 pathway activation is cell non-autonomous and results in global transcriptional changes, some of which may regulate synaptic transmission (Figure 6C). Thus, Nrf family activation may be a conserved mechanism by which stress alters neuronal function during adverse conditions.

### Cell Non-Autonomous Role of SKN-1 on NMJ Function

Our genetic and phenotypic analysis of *wdr-23* mutants supports the idea that the negative regulation of SKN-1 by WDR-23 is required for the normal release of neurotransmitter at NMJs. Interestingly, we found that although WDR-23 was nearly ubiquitously expressed in multiple tissues, WDR-23 acts cell non-autonomously in the intestine to regulate neuronal function, suggesting that increased SKN-1 activity in the intestine negatively regulates synaptic transmission. High copy *skn-1* transgenes expressed in the intestine are toxic [26], and even low copy transgenes make animals sick (data not shown), precluding direct behavioral characterization of SKN-1 over-expressing animals. The intestine is a first line of defense for *C. elegans* in their natural environment. Similarly, analysis of human disease-associated Nrf2 polymorphisms indicate that the organ systems which come in contact with environmental stressors, such as skin, lungs and intestines, are often highly compromised [1]. Thus, detection of environmental stress by the intestine could result in increased intercellular communication between the intestine and nervous system to promote neuronal survival.

Our results raise the possibility that a diffusible factor released from the intestine might mediate the effects of SKN-1 activation on the nervous system. One possible model is that increased SKN-1 activity alters the expression and/or release of neuropeptides from the intestine. Indeed, RNA sequencing indicates that genes involved in neuropeptide signaling in the intestine are up-regulated in *wdr-23* mutants, including neuropeptides, insulins and FMRFamides. Furthermore, our observation that WDR-23 promotes neuropeptide secretion from neurons suggests that neuropeptide secretion from the intestine might also be under WDR-23/SKN-1 regulation. Interestingly, recent work has established signaling from the nervous system to the intestine during activation of mitochondrial unfolded protein response [41], and protons released from the intestine directly affect muscular activity [50]. Together, these studies highlight the importance of endocrine signaling from the intestine in regulating survival and behavior.

### Mitochondrial Function of WDR-23

We show that WDR-23a is primarily localized to mitochondria whereas WDR-23b is primarily nuclear, yet both *wdr-23a* and *wdr-23b* transgenes rescue *wdr-23* mutant phenotypes. Thus specific sub-cellular localization of WDR-23 may not be critical for its function. Alternatively, it is possible that localization of WDR-23 is critical for function, but localized fluorescence of WDR-23-GFP transgenes may be below our limits of detection by fluorescence microscopy.

Nrf2 is proposed to be dynamically associated with the cytosol, nucleus, actin filaments and mitochondria, and its nuclear abundance might be influenced by factors regulating its association with these compartments during stress [1]. Nrf2 is tethered to mitochondria via its interaction with Keap1 and PGAM5 [1], [51], [52], and Keap1 directly detects increased levels of reactive oxygen species released by mitochondria [5], [53], [54]. *C. elegans* do not encode a clear Keap1 ortholog. Thus, WDR-23 might play a homologous role in *C. elegans* to detect increased levels of oxidative stress or mitochondrial damage. We speculate that WDR-23a may associate with mitochondria dynamically and may be released from mitochondria during stress, where it can regulate SKN-1 translocation or degradation. In support of this hypothesis, a recent study has established that SKN-1 interacts with the mitochondrial protein PGAM-5, and inhibition of this interaction results in constitutively activated SKN-1 and a perceived state of starvation [27].

Why might increased levels of SKN-1 result in decreased neuromuscular activity? During stress, organisms might reallocate energy for critical cellular processes by diverting energy consumption away from non-essential processes, such as energy demanding synaptic transmission. Consistent with this prediction, previous work has established that changes in reactive oxygen species can result in altered evoked and spontaneous neurotransmitter release [15]. Furthermore, reduced energy results in the activation of AMP-activated protein kinase, AMPK, which results in increased mitophagy [55] and SKN-1-dependent changes in longevity [28]. Thus, it is interesting to speculate that reduced energy, possibly caused by damaged mitochondria, activates SKN-1, resulting in broad transcriptional changes meant to scavenge oxidants and conserve energy across multiple tissues.

### Stress Regulation of Synaptic Transmission

Our results indicate that not all environmental stressors that activate SKN-1 impact synaptic transmission. We found that arsenite, which has previously been shown to strongly activate *skn-1* [7], [30] robustly induced *skn-1-*dependent aldicarb resistance, whereas other classical *skn-1* activators did not. Thus, arsenite may be a more potent activator of SKN-1. Alternatively, it is possible that different environmental stressors elicit differential SKN-1 dependent transcriptional programs. SKN-1/Nrf2 is regulated by diverse signaling pathways, and Nrf2 differentially associates with multiple cofactors, providing potential mechanisms for stress-specific and cell-type-specific SKN-1/Nrf2 responses [1]. Indeed, microarray analyses of *C. elegans* indicate that different stressors activate distinct but overlapping SKN-1-dependent gene expression profiles [7], [17].

While previous microarray studies have identified approximately 200 genes to be transcriptionally regulated by SKN-1 [7], [17], [27], [47], our RNA sequencing indicates that the expression of over 2000 genes is altered in *wdr-23* mutants. We do not think that the increased number of genes in our study is due to SKN-1 independent functions of *wdr-23*, since *skn-1* mutations fully block all *wdr-23* defects. Instead, this increase is likely due to greater sensitivity of RNA sequencing compared to microarrays, allowing detection of genes with low abundance or restricted tissue distribution [56], [57]. For example, we found the expression of the GPCR signaling genes *odr-3* and *odr-10*, which are both expressed in a few sensory neurons, to be up regulated approximately 6 fold in *wdr-23* mutants. Although our behavioral and phenotypic analyses indicate that the sole function of *wdr-23* in adults is to negatively regulate SKN-1, we cannot exclude the possibility that some of the transcriptional changes detected in *wdr-23* mutants may be SKN-1-independent.

Increases in expression of synaptic transmission genes could explain the reduction in NMJ function in *wdr-23* mutants. *rab-3/*RAB3 expression was increased 3.3 fold in *wdr-23* mutants, for example, and over-expression of Rab3 in adrenal chromaffin cells causes reduced exocytosis [58], [59]. However, given that our results show intestinal SKN-1 regulates synaptic function, it seems likely that these synaptic transmission genes are indirectly regulated by SKN-1, possibly by an endocrine mechanism. Identifying the functionally relevant transcriptional targets that mediate the effects of *wdr-23* on synaptic transmission will be important in understanding regulation of neuronal function by SKN-1.

## Methods

### *C. elegans* Strains

Strains were cultured at 20° using standard methods. All experiments were performed on young adult hermaphrodites unless otherwise indicated. Strains *skn-1(zu67)*, *skn-1(zu135)* and *glo-1(zu391)* were provided by the Caenorhabditis Genetics Center, which is funded by the NIH National Center for Research Resources (NCRR). Strain *wdr-23(tm1817)* was provided by National BioResource Project (Japan). The wild type reference strain was N2 Bristol. The following strains were also used: *drp-1(tm1108)*, *unc-104(e1265)*, *unc-10(e102)*, *nre-1(hd20) lin-15b(hd126)*, *sid-1(qt2)*, *skn-1(lax120)*, *skn-1(lax188)*, EG4322 *ttTi5605;unc-119(ed9)*, PS6268[*Pmyo-3-INVOM-YFP*, *Pmyo-3-MatrixCFP*, *rol-6*], nuIs122[*Pmyo-2-GFP*, *Pacr-2-synaptophluorin*], nuIs152[*Pttx-1-RFP*, *Punc-129-GFP-SNB-1]*II, nuIs321[*Pmyo-2-GFP*, *Punc-17-mCherry*]. All strains were outcrossed at least 6 times. Transgenic lines expressing integrated fluorescence markers in a *skn-1* mutant background resulted in larval arrest, making fluorescence imaging of *skn-1* mutant adults impossible.

### Molecular Biology

The genomic *wdr-23* fragment, *wdr-23* promoter, *ges-1* promoter, and *col-12* promoter were amplified by PCR from mixed-stage genomic DNA. *C. elegans* cDNA was used to clone all genes and truncations into pPD49.26 or pCFJ151 using standard molecular biology techniques. The WDR-23(DxR) mutation was made by QuikChange PCR (Stratagene) targeting the two DxR motifs (R342H, R389H). For a detailed list of all primers used, refer to Text S1.

### Transgenic Lines

Transgenic strains were generated by injecting N2 or *wdr-23(tm1817)* with expression constructs (2.5–25 ng/µL) and the co-injection marker KP#708 (*Pttx-3-rfp*, 40 ng/µL) or KP#1106 (*Pmyo-2-gfp*, 10 ng/µL). Microinjection was performed using standard techniques as previously described [60]. At least three lines for each transgene were examined, and a representative transgene is shown. For a detailed list of all transgenes used, refer to Text S1.

### Locomotion Assays

The body bends assay was completed using age matched young adult animals. Animals were individually moved onto a NGM plate without food and allowed to acclimate for 30 seconds. After 30 seconds, body bends were counted per worm for 60 seconds for at least 30 animals per genotype.

Quantification of time spent in locomotion was completed using age matched young adult animals. Single L4 animals were moved onto freshly seeded OP50 NGM plates and incubated overnight at 20°. The next day, at least 20 animals per genotype were evaluated for time spent in forward locomotion. To induce locomotion, the lid of the plate was removed and quickly replaced. This generally resulted in a quick bout of backward locomotion followed by forward locomotion. Quantification began once the animal resumed forward locomotion. Time was scored using Ethotimer software (Thomas Lab, University of Washington).

### Pharmacology

For analysis of sensitivity to the drugs aldicarb and levamisole, paralysis of adult worms was scored every 10 or 15 minutes, starting at 40 minutes, using 1.0 mM aldicarb (Bayer) or 200 µM levamisole (Sigma) unless otherwise specified. For experiments, three replicates of at least 20 worms per genotype were placed on NGM plates supplemented with paralytic, and the number of worms paralyzed on each plate was counted to calculate the percent paralyzed at each time point per genotype. The percentages were averaged at each time point per genotype and plotted graphically. For each experiment, the genotype was blind to the scorer, and the analysis was repeated at least two times. Results were analyzed using GraphPad Prism 5 software, and significances were calculated by one-way repeated measures ANOVA with Tukey's test for multiple comparisons.

For pretreatment with sodium arsenite (RICCA Chemicals), age matched L4 animals were placed on NGM plates supplemented with 1.0 or 2.0 mM sodium arsenite. Animals were incubated at 20° for 14 hours, then moved to NGM without arsenite for recovery for 3 hours prior to aldicarb assay.

### Microscopy and Analysis

Fluorescence microscopy experiments were performed as previously described [33]. Our preliminary studies indicated expression of *unc-129*, the DA motor neuron promoter used by Ch'ng et al., 2008, increased modestly in a *skn-1* dependent manner in *wdr-23* mutants (data not shown). Therefore, we used the *unc-17* promoter for all of our quantitative imaging. Expression of *unc-17* does not change in *wdr-23* mutants according to an *unc-17* reporter construct, qRT-PCR or RNAseq (Figure 3B, Table 3 and Table S4). To image animals, L4 or adult worms were paralyzed using 2,3-butanedione monoxime (BDM, 30 µg/µL; Sigma) and mounted on 2% agarose pads for imaging. Images of synapses were captured from dorsal axons of DA neurons near the posterior gonadal bend of the worm. At least 30 worms were imaged for all quantitative experiments. For all fluorescence microscopy experiments, images were captured with a Nikon eclipse 90i microscope equipped with a Nikon PlanApo 60× or 100× objective (NA = 1.4) and a Photometrics Coolsnap ES2 camera. Metamorph 7.0 software (Universal Imaging/Molecular Devices) was used to capture serial image stacks, and the maximum intensity projection was used for analysis of the dorsal cords. Line scans of the maximum intensity projection image were also recorded using Metamorph. The fluorescence intensity values were then quantified using Puncta 6.0 software written with Igor Pro (Wavemetrics), as previously described [33], [34]. For all experiments, fluorescence values were normalized to the values of 0.5 µm FluoSphere beads (Invitrogen) captured during each imaging session. This was performed to provide a standard for comparing absolute fluorescence levels between animals from different sessions.

### RNA Interference

*Feeding RNAi:* Feeding RNAi was completed as described [61]. Briefly, animals were synchronized by a hypochloride/sodium hydroxide egg preparation and placed on RNAi plates containing HT1115(DE3) bacteria specific for *unc-17*, *wdr-23*, or the empty vector L4440 from the Ahringer library [61]. Aldicarb analysis was completed three or four days after the egg preparation.

*Hairpin RNAi:* The inverted repeat hairpin targeting *wdr-23* was constructed as described [62] and injected directly into *sid-1(qt2)* mutants. Multiple lines were analyzed for each injection.

### Whole-Genome Sequencing

From an ethyl methanesulfonate (EMS) screen to identify genes that suppress the small size and aldicarb response of *wdr-23* mutants, we isolated the allele *vj24*. DNA was extracted using Qiagen Gentra Purgene system from mixed staged *wdr-23;vj24* heterozygous animals outcrossed three times. Sequencing was completed in single reads of 101 bp using an Illumnia HiSeq2000 with HiSeqv3 flow cell at an average sequencing depth of 38. Data was analyzed using MAQGene [63].

### RNA Sequencing

Total RNA was isolated from approximately 10,000 mixed stage animals for wild type and *wdr-23* mutants using Stat60 (Tel-test B, Texas). Transcriptome libraries were prepared using TruSeq RNA sample preparation kit (Illumina) according to manufacturer's TruSeq protocol rev. A. In brief, polyadenylated mRNA was isolated from 0.1–4 ug of total RNA using oligo(dT) beads, fragmented, first strand cDNA synthesized using random primers and SuperScript II reverse transcriptase (Invitrogen) followed by synthesis of a second strand. Ends of resulting double-stranded fragments were repaired, 3′ ends were adenylated and adapters were ligated to the fragment, completing the library preparation. Library then was amplified by PCR and quality and quantity of libraries were evaluated on BioAnalyzer 2100 (Agilent). Sequencing was performed on HiSeq2000 (Illumina). Sequencing reads were aligned to the *C. elegans* genome (release WS210) using TopHat [45]. Libraries were sequenced to a depth of 36.1 and 34.9 million reads for wild type and *wdr-23* mutants, respectively. Gene models were downloaded from ENSEMBL and quantified using Cufflinks. Differentially expressed genes at false discovery rate (FDR) of 0.05 were identified using the Cuffdiff module of the Cufflinks package.

### qRT–PCR

cDNA was synthesized from total RNA using ProtoScript M-MuLV RT-PCR (NEB). SYBR Premix Ex Taq (Clontech) was used in an BioRad Chromo4 Real-Time PCR Detector in triplicate. Data was analyzed using the comparative *C_t_* method. Relative mRNA levels were normalized to *rpl-2*, and calculated from at least two biological replicates. Primers were designed to be intron-spanning.

## Supporting Information

Figure S1 Illustration of the growth delay and size differences in *wdr-23* mutants relative to wild-type controls. (A) Confocal images of young adult wild type and *wdr-23* mutants expressing GFP under the *acr-2* promoter, showing that motor neuron patterning is similar in *wdr-23* mutants and that *wdr-23* mutants are smaller than wild type controls. (B) Percent of animals at each developmental stage for wild type (n = 575), *wdr-23*(*tm1817*, n = 364), *skn-1*(*zu67*, n = 108), and *skn-1;wdr-23* (n = 95) mutants at 27, 43, and 52 hours after egg lay. For egg lay, adult animals were allowed to lay eggs for exactly 3 hours on freshly seeded NGM plates. (C) Worm length of two-day-old animals for wild type (n = 85, *wdr-23*(*tm1817*, n = 93), *skn-1*(*zu67*, n = 98), *skn-1;wdr-23* (n = 86) and genomic rescue (n = 74). Genomic rescue indicates a genomic *wdr-23* fragment expressed in *wdr-23* mutants. Animals were picked as L4s to age match genotypes, then allowed to incubate at 20° for 48 hours prior to scoring. Images were captured by Metamorph 7.0, and linescans were drawn down the length of the animals to calculate body length (ANOVA, followed by Tukey's post-hoc). (Scale bar represents 100 µm; ***p<0.001.)
(TIF)

Figure S2 WDR-23(DxR) has wild type localization. Fluorescence images showing localization of WDR-23a-GFP and WDR-23(DxR)-mCherry in motor neuron axons (driven by the *unc-129*). (Scale bar represents 10 µm.)
(TIF)

Figure S3 Expression pattern of *wdr-23b*. Representative images showing expression pattern of a 2.0 kb *wdr-23b* promoter fragment tagged to a nuclear localized *gfp* in indicated tissues. Box highlights ring-like structures reminiscent to those seen by full length WDR-23a-GFP. The *wdr-23b* promoter fragment contains the first two exons of *wdr-23a*; removal of these exons (in the WDR-23(repeats)-GFP construct) results in uniform GFP throughout the cell. Thus, these two exons of *wdr-23a* target the protein to mitochondria. (Scale bar represents 10 µm.)
(TIF)

Figure S4 Intestinal outer mitochondrial membrane. Fluorescence images showing distribution of an inverted outer membrane mitochondrial marker in the intestine (*Pges-1-*INVOM-RFP). Green image shows gut autofluorescence. (Scale bar represents 10 µm.)
(TIF)

Table S1 Quantification and statistical analysis of locomotion assays.
(PDF)

Table S2 Analysis of fluorescence markers in *wdr-23* mutants. WDR-23a and WDR-23b indicate *wdr-23* rescue with respective cDNA driven by the endogenous *wdr-23* promoter and integrated via single copy insertion. (Student's *t-*test; *p<0.05, **p<0.01, ***p<0.001.)
(PDF)

Table S3 SKN-1 consensus sequence analysis. SKN-1 consensus sequence analysis of all up-regulated genes, stress/detox genes, behavior genes, and down-regulated genes in *wdr-23* mutants compared to a randomized list of 1200 genes which do not statistically change in *wdr-23* mutants relative to wild type controls. Analysis was completed using promoter fragments containing either 1000 bp or 500 bp upstream of the transcriptional start as determined by RSAT. ‘Occurrences’ indicates the total number of genes with at least one consensus site.
(PDF)

Table S4 RNA-seq list of genes up-regulated in *wdr-23* mutants. Genes are classified based on InterPro domain and GO term from DAVID analysis. Ref column indicates genes previously established to be up-regulated by SKN-1 using microarray studies with the following: *skn-1* RNAi^1^, exposure to arsenite^2^, exposure to *t-*butyl hydrogen peroxide^3^ [7] or hyperbaric oxygen^4^ [17].
(XLS)

Table S5 Genes potentially involved in NMJ function increased in *wdr-23* mutants. Genes with likely roles in regulating synaptic function or behavior identified by RNA-seq. Genes are classified based on InterPro domain and GO term from DAVID analysis as in Table S4. Expression indicates tissue expression identified by www.wormbase.org. Neuronal^+^ indicates in neurons as well as other tissue types.
(XLS)

Table S6 RNA-seq list of genes down-regulated in *wdr-23* mutants. Genes are classified based on InterPro domain and GO term from DAVID analysis. Ref column indicates genes previously established to be negatively regulated by *skn-1* RNAi [7].
(XLS)

Text S1 Complete list of primers and transgenes. List of all primers and injected transgenes used in the study.
(PDF)
