# Supplementary figures and images for: The Conserved SKN-1/Nrf2 Stress Response Pathway Regulates Synaptic Function in Caenorhabditis elegans
Source: PLoS Genet. 2013 Mar 21;9(3):e1003354. doi: 10.1371/journal.pgen.1003354 (PMC3605294; doi:10.1371/journal.pgen.1003354)

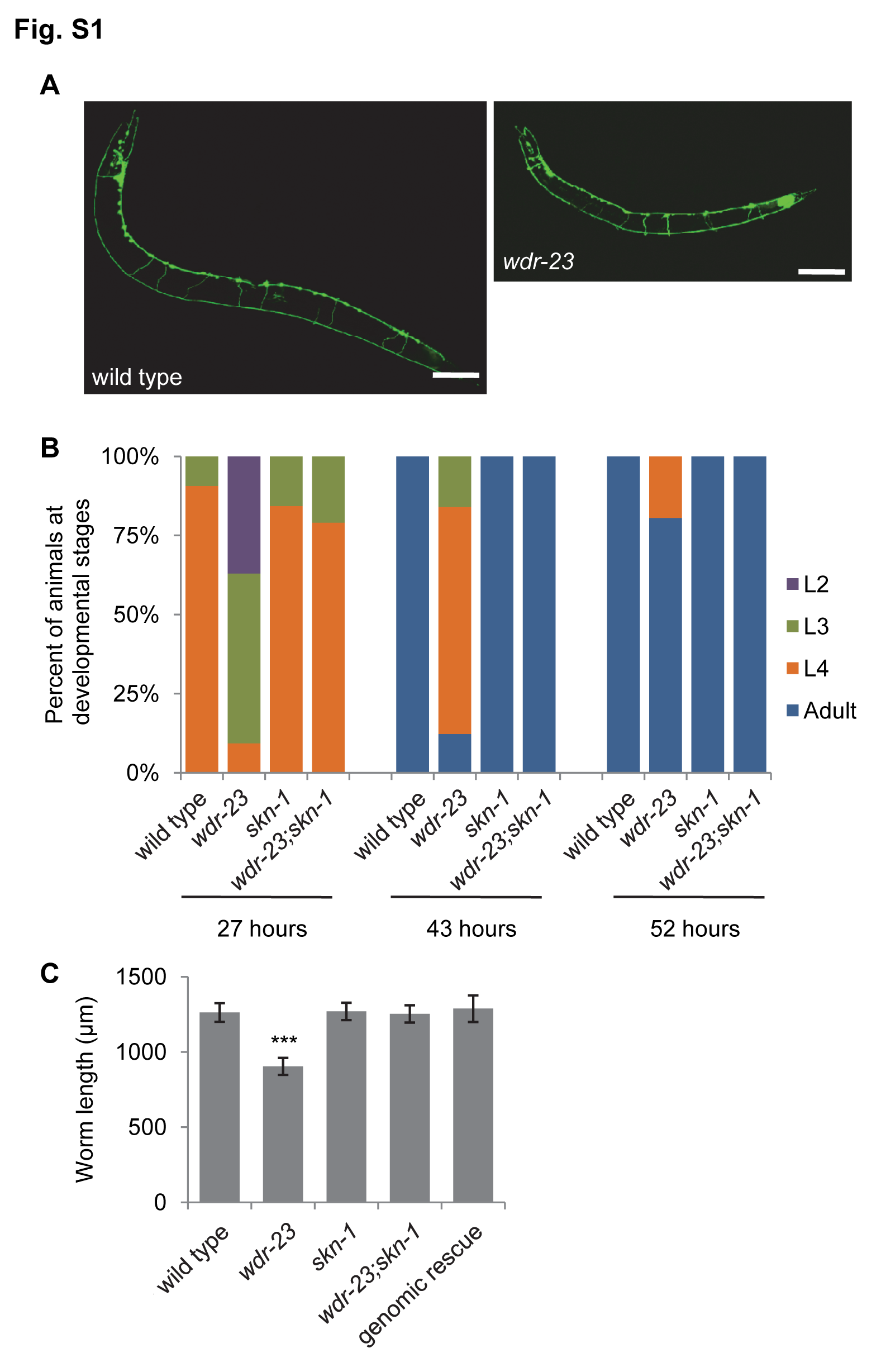

Supplement: Figure S1 — Illustration of the growth delay and size differences in wdr-23 mutants relative to wild-type controls. (A) Confocal images of young adult wild type and wdr-23 mutants expressing GFP under the acr-2 promoter, showing that motor neuron patterning is similar in wdr-23 mutants and that wdr-23 mutants are smaller than wild type controls. (B) Percent of animals at each developmental stage for wild type (n = 575), wdr-23(tm1817, n = 364), skn-1(zu67, n = 108), and skn-1;wdr-23 (n = 95) mutants at 27, 43, and 52 hours after egg lay. For egg lay, adult animals were allowed to lay eggs for exactly 3 hours on freshly seeded NGM plates. (C) Worm length of two-day-old animals for wild type (n = 85, wdr-23(tm1817, n = 93), skn-1(zu67, n = 98), skn-1;wdr-23 (n = 86) and genomic rescue (n = 74). Genomic rescue indicates a genomic wdr-23 fragment expressed in wdr-23 mutants. Animals were picked as L4s to age match genotypes, then allowed to incubate at 20° for 48 hours prior to scoring. Images were captured by Metamorph 7.0, and linescans were drawn down the length of the animals to calculate body length (ANOVA, followed by Tukey's post-hoc). (Scale bar represents 100 µm; ***p<0.001.) (TIF) [file pgen.1003354.s001.tif]

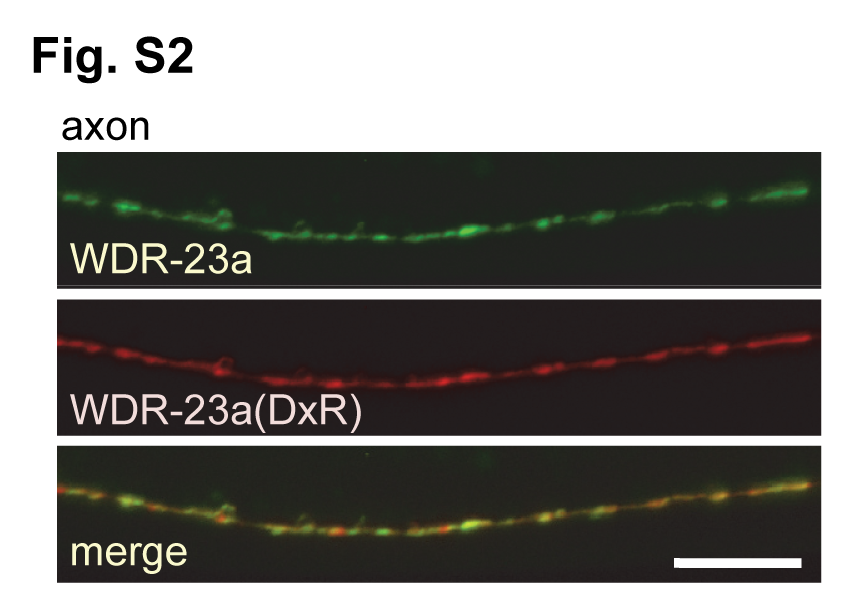

Supplement: Figure S2 — WDR-23(DxR) has wild type localization. Fluorescence images showing localization of WDR-23a-GFP and WDR-23(DxR)-mCherry in motor neuron axons (driven by the unc-129). (Scale bar represents 10 µm.) (TIF) [file pgen.1003354.s002.tif]

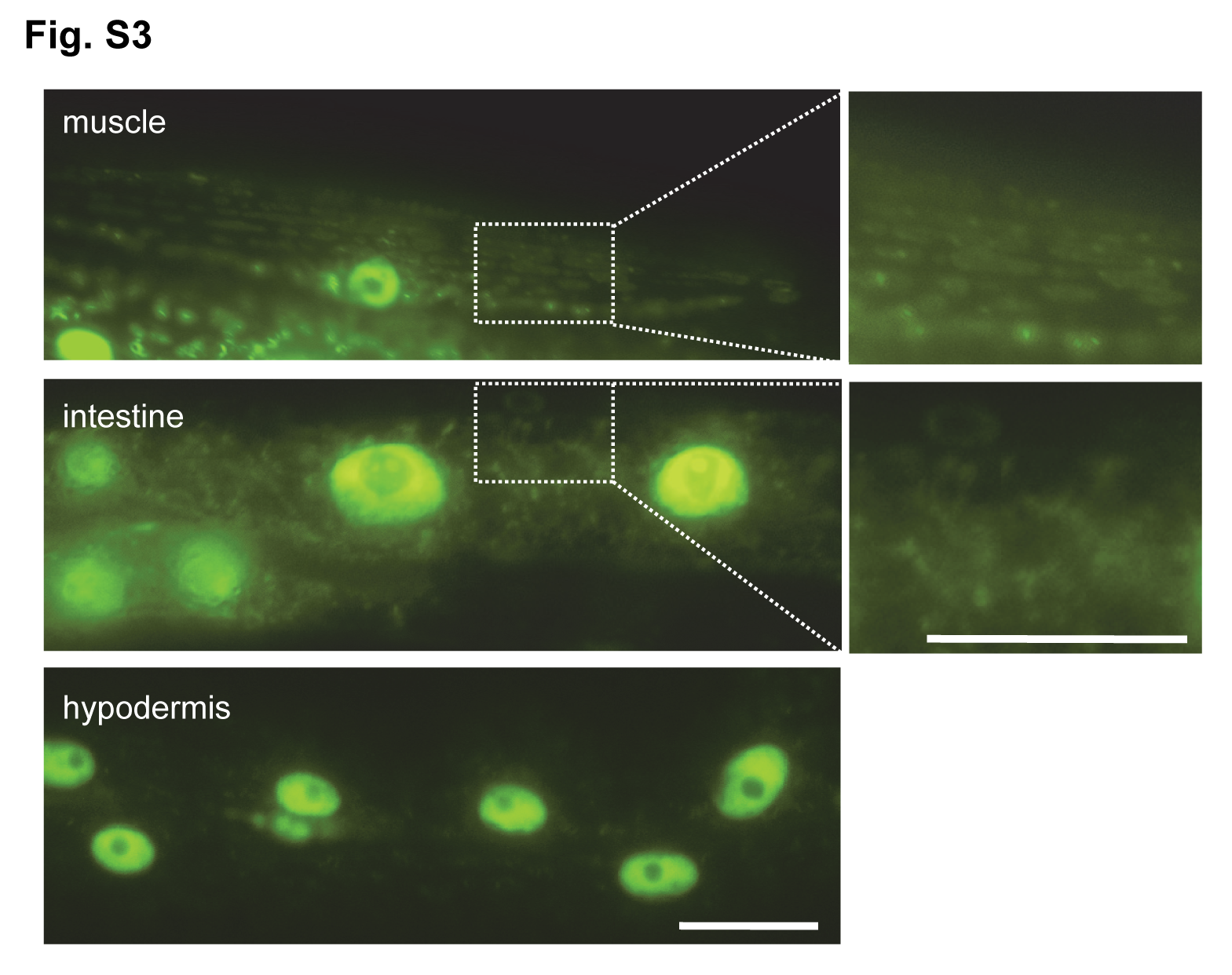

Supplement: Figure S3 — Expression pattern of wdr-23b. Representative images showing expression pattern of a 2.0 kb wdr-23b promoter fragment tagged to a nuclear localized gfp in indicated tissues. Box highlights ring-like structures reminiscent to those seen by full length WDR-23a-GFP. The wdr-23b promoter fragment contains the first two exons of wdr-23a; removal of these exons (in the WDR-23(repeats)-GFP construct) results in uniform GFP throughout the cell. Thus, these two exons of wdr-23a target the protein to mitochondria. (Scale bar represents 10 µm.) (TIF) [file pgen.1003354.s003.tif]

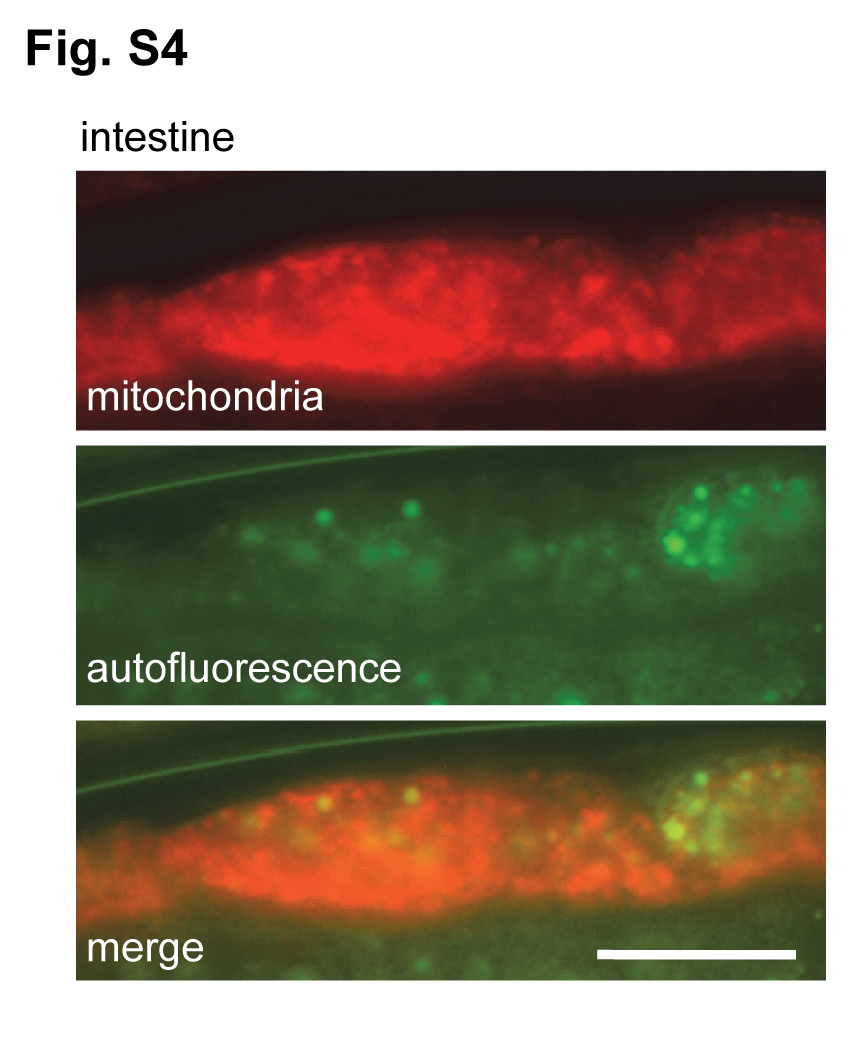

Supplement: Figure S4 — Intestinal outer mitochondrial membrane. Fluorescence images showing distribution of an inverted outer membrane mitochondrial marker in the intestine (Pges-1-INVOM-RFP). Green image shows gut autofluorescence. (Scale bar represents 10 µm.) (TIF) [file pgen.1003354.s004.tif]
