# Supplementary material for: The Conserved SKN-1/Nrf2 Stress Response Pathway Regulates Synaptic Function in Caenorhabditis elegans
Source: PLoS Genet. 2013 Mar 21;9(3):e1003354. doi: 10.1371/journal.pgen.1003354 (PMC3605294; doi:10.1371/journal.pgen.1003354)
Supplement: Table S1 — Quantification and statistical analysis of locomotion assays. (PDF) [file pgen.1003354.s005.pdf]

Table S1. Locomotion assays

Fig. 1B, Body bends per minute

genotype	n	bends	*p* value
wild type	30	36.8 ± 0.9	
wdr-23	30	30.5 ± 1.1	<0.001
WDR-23 rescue	30	35.2 ± 0.7	0.17

Fig. 4C, Percent paralysis

genotype	N	percent	*p* value
control	62	80 ± 7%	
Pwdr-23	71	45 ± 3%	0.003
Prab-3 (neuron)	78	74 ± 1%	0.317
Pges-1 (intestine)	71	41 ± 5%	0.003
Pmyo-3 (muscle)	65	61 ± 8%	0.064
