# Supplementary material for: The Conserved SKN-1/Nrf2 Stress Response Pathway Regulates Synaptic Function in Caenorhabditis elegans
Source: PLoS Genet. 2013 Mar 21;9(3):e1003354. doi: 10.1371/journal.pgen.1003354 (PMC3605294; doi:10.1371/journal.pgen.1003354)
Supplement: Table S2 — Analysis of fluorescence markers in wdr-23 mutants. WDR-23a and WDR-23b indicate wdr-23 rescue with respective cDNA driven by the endogenous wdr-23 promoter and integrated via single copy insertion. (Student's t-test; *p<0.05, **p<0.01, ***p<0.001.) (PDF) [file pgen.1003354.s006.pdf]

Table S2. Analysis of fluorescent markers

Figure 3: Punctal analysis				
RAB-3/Rab3	wild type, n=35	*wdr-23*, n=33	WDR-23a, n=31	WDR-23b, n=33
peak fluorescence (arb. units)	366.77 ± 35.41	681.21 ± 77.46***	364.37 ± 41.51	482.74 ± 35.48
cord fluorescence (arb. units)	106.96 ± 11.28	150.57 ± 24.15	88.77 ± 12.64	138.60 ± 13.16
punctal width (µm)	1.36 ± 0.04	1.35 ± 0.05	1.38 ± 0.04	1.45 ± 0.05
interpunctal interval (µm)	2.70 ± 0.05	2.63 ± 0.07	2.70 ± 0.05	2.74 ± 0.09
SNB-1/synatobrevin	wild type, n=40	*wdr-23*, n=38		
peak fluorescence (arb. units)	428.22 ± 13.10	578.69 ± 20.71***		
cord fluorescence (arb. units)	143.45 ± 6.12	136.65 ± 7.16		
punctal width (µm)	1.61 ± 0.03	1.61 ± 0.04		
interpunctal interval (µm)	2.77 ± 0.05	2.64 ± 0.05*		
INS-22	wild type, n=34	*wdr-23*, n=36	WDR-23a, n=34	WDR-23b, n=30
peak fluorescence (arb. units)	408.06 ± 16.58	669.87 ± 26.54***	468.68 ± 24.05*	407.28 ± 20.74
cord fluorescence (arb. units)	41.21 ± 3.58	56.72 ± 7.79	40.59 ± 3.86	33.72 ± 4.04
punctal width (µm)	0.98 ± 0.03	1.10 ± 0.03**	1.04 ± 0.03	0.94 ± 0.02
interpunctal interval (µm)	2.03 ± 0.032	2.16 ± 0.03**	2.07 ± 0.03	2.07 ± 0.03
NLP-21	wild type, n=30	*wdr-23*, n=30		
peak fluorescence (arb. units)	338.74 ± 29.08	572.07 ± 35.08***		
cord fluorescence (arb. units)	23.72 ± 3.07	41.28 ± 8.37		
punctal width (µm)	1.02 ± 0.03	1.00 ± 0.03		
interpunctal interval (µm)	2.14 ± 0.04	2.10 ± 0.04		
NLP-21	wild type, n=33	*wdr-23,* n=35		
coelomocytes (arb. units)	1180.89 ± 92.74	755.55 ± 68.06***		
UNC-10/Rim1	wild type, n=31	*wdr-23*, n=31		
peak fluorescence (arb. units)	186.43 ± 23.42	188.69 ± 24.09		
cord fluorescence (arb. units)	13.26 ± 2.92	15.10 ± 2.26		
punctal width (µm)	0.75 ± 0.03	0.72 ± 0.02		
interpunctal interval (µm)	2.01 ± 0.06	1.99 ± 0.08		
Soluble mCherry	wild type, n=30	*wdr-23*, n=30		
peak fluorescence (arb. units)	1555.85 ± 70.38	166.33 ± 74.97		
cord fluorescence (arb. units)	652.00 ± 69.11	783.95 ± 60.95		
punctal width (µm)	1.83 ± 0.05	1.76 ± 0.04		
interpunctal interval (µm)	3.28 ± 0.15	3.13 ± 0.10		
Signal sequence GFP	wild type, n=29	*wdr-23*, n=31		
coelomocytes (arb. units)	2105.9 ± 130.51	1975.17 ± 130.19		
