# Supplementary material for: The Conserved SKN-1/Nrf2 Stress Response Pathway Regulates Synaptic Function in Caenorhabditis elegans
Source: PLoS Genet. 2013 Mar 21;9(3):e1003354. doi: 10.1371/journal.pgen.1003354 (PMC3605294; doi:10.1371/journal.pgen.1003354)
Supplement: Table S3 — SKN-1 consensus sequence analysis. SKN-1 consensus sequence analysis of all up-regulated genes, stress/detox genes, behavior genes, and down-regulated genes in wdr-23 mutants compared to a randomized list of 1200 genes which do not statistically change in wdr-23 mutants relative to wild type controls. Analysis was completed using promoter fragments containing either 1000 bp or 500 bp upstream of the transcriptional start as determined by RSAT. ‘Occurrences’ indicates the total number of genes with at least one consensus site. (PDF) [file pgen.1003354.s007.pdf]

Table S3. Promoter analysis for SKN-1 consensus sequences

		1000bp promoter		500bp promoter	
		WWTDTCAT	TTDTCATC	WWTDTCAT	TTDTCATC
Up-regulated (2285)	Occurrences	1826	670	1323	426
	Percentage	79.9%	29.3%	57.9%	18.6%
Up-regulated (top 500)	Occurrences	431	202	335	143
	Percentage	86.2%	40.4%	67.0%	28.6%
Up-regulated (top 300)	Occurrences	271	133	212	102
	Percentage	90.3%	44.3%	70.7%	34.0%
Down-regulated (134)	Occurrences	106	23	83	17
	Percentage	79.1%	17.2%	61.9%	12.7%
Randomized list (1200)	Occurrences	943	305	674	169
	Percentage	78.6%	25.4%	56.2%	14.1%
Behavior (165)	Occurrences	135	36	102	27
	Percentage	81.8%	21.8%	61.8%	16.4%
Stress/detox (165)	Occurrences	141	85	109	63
	Percentage	85.5%	51.5%	66.1%	38.2%
