# Supplementary material for: The Conserved SKN-1/Nrf2 Stress Response Pathway Regulates Synaptic Function in Caenorhabditis elegans
Source: PLoS Genet. 2013 Mar 21;9(3):e1003354. doi: 10.1371/journal.pgen.1003354 (PMC3605294; doi:10.1371/journal.pgen.1003354)
Supplement: Text S1 — Complete list of primers and transgenes. List of all primers and injected transgenes used in the study. (PDF) [file pgen.1003354.s011.pdf]

Supplemental Text S1

Oligos used for molecular cloning

<u>sequence</u>	<u>primer</u>
genomic *wdr-23*	5'-tcgtcgAAGCTTggaaaataaacgaagctgccg-3'
	5'-ttccctcatttcaagagac-3'
wdr-23 promoter	5'-tcgtcgAAGCTTggaaaataaacgaagctgccg-3'
	5'-tcgtcgGCATGCtcgagcatagggcgaagcac-3'
WDR-23 inverted repeat hairpin	5'-ccccccGCTAGCaaaaatgggcaactggataacgtcgacg-3'
	5'-ccccccACCGGTgatttgttcactgctatccg-3'
	5'-ccccccGGTACCaaaaatgggcaactggataacgtcg-3'
	5'-ccccccACCGGTttgaaggtctcgattgaggatg-3'
WDR-23a	5'-ccccccGCTAGCaaaaatgggcaactggataacgtcgacg-3'
	5'-ccccccGGTACCttaattttgagagatgctgctcg-3'
WDR-23b	5'- ccccccGCTAGCaaaaatgccttacaaaagacattcc-3'
	5'-ccccccGGTACCttaattttgagagatgctgctcg-3'
WDR-23(repeat)	5'-ccccccGCTAGCaaaaatgaatcacagcaatgacagtgacac-3'
	5'-ccccccACCGGTattttgagagatgctgctcgatgagc-3'
DxR motif (R342H)	5'-gtaaaggtatgggacaaacatgcatggtcagatggagac -3'
	5'-gtctccatctgaccatgcatgtttgtcccatacctttac-3'
DxR motif (R389H)	5'- caatcaaagtttgggatcttcataagttttcg-3'
	5'- cctgacatattcgaaaacttatgaagatccc-3'
col-12 promoter (0.4kb)	5'-tcgtcgGCATGCattttcagtatttgctattgac-3'
	5'-tcgtcgGGATCCttttctaaaaagtaatcaaatc-3'
ges-1 promoter (2.0kb)	5'-ccccccGCATGCaactccgaactatgatgacg-3'
	5'-ccccccGGATCCctgaattcaaagataagatatgtaatag-3'
rab-3 promoter (1.5kb)	5'-tgctcgGCATGCatcttcttggttcaatttc-3'
	5'-tgctcgGGATCCctgaaaatagggctactgtag-3'
gst-4 qRT-PCR	5'-aagctgaagccaacgactc-3'
	5'-gcttcttcctctgcagtttttc-3'
gst-30 qRT-PCR	5'-agtcgactatgccgttcg-3'
	5'-tggacatcttccatttattcca-3'
rpl-2 qRT-PCR	5'-tgtggagctaaggctcaaatc-3'
	5'-cgaatacgtgtcttcttggtgt-3'
pmp-3 qRT-PCR	5'-gttcccgtgttcatcactcat-3'
	5'-acaccgtcgagaagctgtaga-3'
cdc-42 qRT-PCR	5'-ctgctggacaggaagattacg-3'
	5'-ctcggacattctcgaatgaag-3'
snt-1 qRT-PCR	5'-cagtaaagcaaatagcaacaacgac-3'
	5'-aaatacgaacgcccattcag-3'
unc-25 qRT-PCR	5'-tgctttactagcgaggatagtcac-3'
	5'-atcggttggaatgttgaagc-3'
unc-17 qRT-PCR	5'-ggttcaaggaaccccaatg-3'
	5'-gagtgtctggcatcatttcg-3'

List of transgenes

Arrays	Plasmid	Construct
vjEx594		genomic wdr-23
vjEx336	pTS59	Pwdr-23-wdr-23a-gfp
	pTS60	Pwdr-23-wdr-23b-gfp
vjEx562	pTS116	Punc-17-unc-10-gfp
vjEx399	pTS63	Punc-17-gfp-rab-3
vjEx334	pTS64	Pges-1-wdr-23a-gfp
	pTS65	Pges-1-wdr-23b-gfp
vjEx418	pDS237	Psnb-1-wdr-23a-gfp
	pTS85	Psnb-1-wdr-23b-gfp
vjEx508	pTS97	Prab-3-wdr-23a-gfp
	pTS98	Prab-3-wdr-23b-gfp
vjEx568	pTS123	Pcol-12-wdr-23a-gfp
	pTS125	Pcol-12-wdr-23b-gfp
vjEx512	pTG3	Pmyo-3-wdr-23a-gfp
	pTG32	Pmyo-3-wdr-23b-gfp
vjEx549	pTS119	Pmyo-3-wdr-23(repeats)-mCherry
vjEx663	pTS147	Punc-129-INVOM-rfp
vjEx339	pDS139	Punc-129-mCherry-snb-1
vjEx237	pTG73	Pwdr-23-wdr-23a(DxR)-GFP
vjEx262	pTG49	Punc-129-wdr-23a(DxR)-mCherry
vjEx423	pDS237	Psnb-1-wdr-23a-gfp
vjEx426	pTS85	Psnb-1-wdr-23b-gfp
vjEx569	pTS126	Pwdr-23-sphk-1(kinase)-gfp
vjEx553	pTS112	Pmyo-3-wdr-23b-mCherry
vjEx664	pTS164	Pnlp-40-wdr-23a-gfp
vjEx665	pTS165	Pnlp-40-wdr-23b-gfp
vjEx565	pTS121	Pwdr-23-wdr-23(repeats)-mCherry
vjEx661	pTS146	Pges-1-INVOM-rfp
vjEx697	pTS167	Pwdr-23-wdr-23(hairpin)
vjEx699	pTS168	Prab-3-wdr-23(hairpin)
vjEx701	pTS169	Pges-1-wdr-23(hairpin)
vjEx703	pTS170	Pnlp-40-wdr-23(hairpin)
vjEx705	pTS171	Pcol-12-wdr-23(hairpin)
vjEx707	pTS172	Pmyo-3-wdr-23(hairpin)
vjEx727	pTS185	Punc-17-ss-gfp

Integrants	Plasmid	Construct	Chromosome
vjIs26	pDS230	Punc-129-wdr-23a-gfp	III
vjIs28	pDS268	Punc-17-ins-22-yfp	X
vjIs30	pDS269	Punc-17-nlp-21-yfp	I
vjIs61	pTS62	Punc-17-gfp-snb-1	V
vjIs74	pTG3	Pmyo-3-wdr-23a-gfp	X
vjIs84	pDS330	Pmyo-3-INVOM-rfp	X
vjIs89	pTS134	Pwdr-23-wdr-23a	II
vjIs90	pTS135	Pwdr-23-wdr-23b	II
